# Mechanochemical Alloying of Molecular Dopants in Expanded Analogs of Halide Perovskites

**DOI:** 10.1021/acs.inorgchem.5c02436

**Published:** 2025-08-20

**Authors:** Xavier Rodriguez-Rubio, Giovanni Vescio, Joshua D. Forero, Albert Cirera, Horst Puschmann, Eliseo Ruiz, Sergi Hernández, Roc Matheu

**Affiliations:** † Department of Inorganic and Organic Chemistry, Institut de Química Teòrica i Computacional, 16724 Universitat de Barcelona, Carrer Martí i Franquès 1, 08028 Barcelona, Spain; ‡ Department of Electronic and Biomedical Engineering, Institute of Nanoscience and Nanotechnology, Universitat de Barcelona, Carrer Martí i Franquès 1, 08028 Barcelona, Spain; § Department of Electronic Engineering, Universitat Politècnica de Catalunya, Carrer Jordi Girona, 1, Edifici C4, 08034 Barcelona, Spain; ∥ Department of Chemistry, 3057 University of Durham, South Road, Durham DH1 3LE, U.K.

## Abstract

Controlling electronic doping remains a fundamental challenge for halide perovskite semiconductors. Introducing molecular dopants in the bulk of the semiconductor is a promising strategy to achieve reliable doping. Herein, we report on a solid-state alloying method that allows the incorporation of molecular dopants with low-lying lowest-unoccupied molecular orbital (LUMO) energy into the bulk of the semiconductor while enabling the fabrication of films by inkjet printing. First, we report on the synthesis and single-crystal structure of three new expanded analogs of halide perovskite (Hepm)­[B_2_X_6_] (B–X = Pb–Br, Pb–I, Sn–I). The cavities in the expanded perovskite analogs are occupied by Hepm^2+^ cations (1-ethylpyrimidin-1,3-diium). The cations are structurally analogous to the Hepz^2+^ cations (1-ethylpyrazin-1,4-diium) but with higher LUMO energy (>0.5 eV). By alloying two expanded lattices via ball-milling, we obtain (Hepz)_*x*_(Hepm)_1–*x*_[Pb_2_Br_6_] and (Hepz)_*x*_(Hepm)_1–*x*_[Pb_2_Br_6*x*_I_6–6*x*_]. The alloyed lattices contain a mixture of molecular dopants with high (Hepm^2+^) and low (Hepz^2+^) LUMO energy, presenting optical gaps as low as 1.25(5) eV. Thus, the solid-state method allows the introduction of Hepz^2+^ cations in the Pb–I lattice, which remained unattained via solution methods. Finally, we also prepare pinhole-free (Hepm)­[Pb_2_Br_6_] films via inkjet printing, proving that expanded perovskites containing molecular dopants are printable.

## Graphical abstract

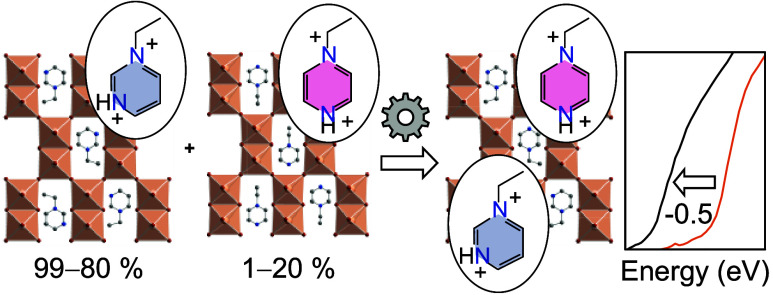

## 1 Introduction

Most semiconductor technologies rely on the intentional engineering of charge transport.^1^ In the case of halide perovskites–a semiconductor class currently developed for critical components in solar cells and light-emitting devices– ^2–6^ reliable control of their charge transport is expected to enhance the performance of optoelectronic devices.^7–9^ Most strategies to dope halide perovskites have attempted to generate intrinsic defects by creating metal-rich/poor or halogen-rich/poor conditions during synthesis,^10–13^ or by introducing impurities (e.g., Na, Bi).^14–17^ Both strategies have resulted in relatively low carrier concentrations and scarce control of the doping type.^11^ Another strategy is charge-transfer doping, exchanging carriers between the perovskite and an external acceptor/donor, such as redox-active molecules.^11,18^ Although charge-transfer doping has recently resulted in significant variation of the charge-carrier concentration,^19–23^ the strategy is limited to the surface of crystallites or grain boundaries, limiting the achievable carrier concentration and rendering dopants prone to surface-initiated degradation.^24^

Incorporating redox-active molecular dopants in the *bulk* of the perovskite structure can overcome the problems inherent to superficial charge-transfer doping while providing a reliable concentration of holes and electrons.^25^ For p-doping, the molecule’s lowest unoccupied molecular orbital (LUMO) must lie close to the semiconductor’s valence band maximum (VBM) to trigger charge-transfer and increase the hole concentration in the semiconductor.^11^ The corner-sharing BX_6_^n‑^ octahedra of the archetypical ABX_3_ perovskites (e.g., CsPbBr_3_, Figure 1A) create a small cavity typically occupied by relatively small cations (e.g., Cs^+^). The atomic orbitals of Cs^+^–or the LUMO of CH_3_NH_3_^+^ in (CH_3_NH_3_)­PbBr_3_–possess energies far from the CBM energy. Thus, most A-sites (e.g., Cs^+^, CH_3_NH_3_^+^) are unable to promote charge-transfer doping in the ABX_3_ perovskites. Expanded analogs of halide perovskites have recently shown that dications with a wide range of LUMO energies can be incorporated as part of their 3D structure. Expanded halide perovskites consist of 3D inorganic sublattices in which B_2_X_10_^*n*–^ dimers (Figure 1B) conceptually replace the BX_6_^*n*–^ octahedra of ABX_3_ halide perovskites (Figure 1A).^26–29^ The expanded A-site cavity allows the incorporation of organic dications with a low-lying LUMO (Figure 1B). Protonated alkyl pyrazinium derivatives, such as H­(Rpz)^2+^ (pz = pyrazinium and R = methyl, ethyl, isopropyl), are the cations with the lowest LUMO energy, such as those comprised in (Hepz)­[Pb_2_Br_6_] (Figure 1B), which display an energy difference between the VBM and the first unoccupied cation-based band (optical gap, *E*_g_) of 1.7 eV.^26^

**Figure 1:**
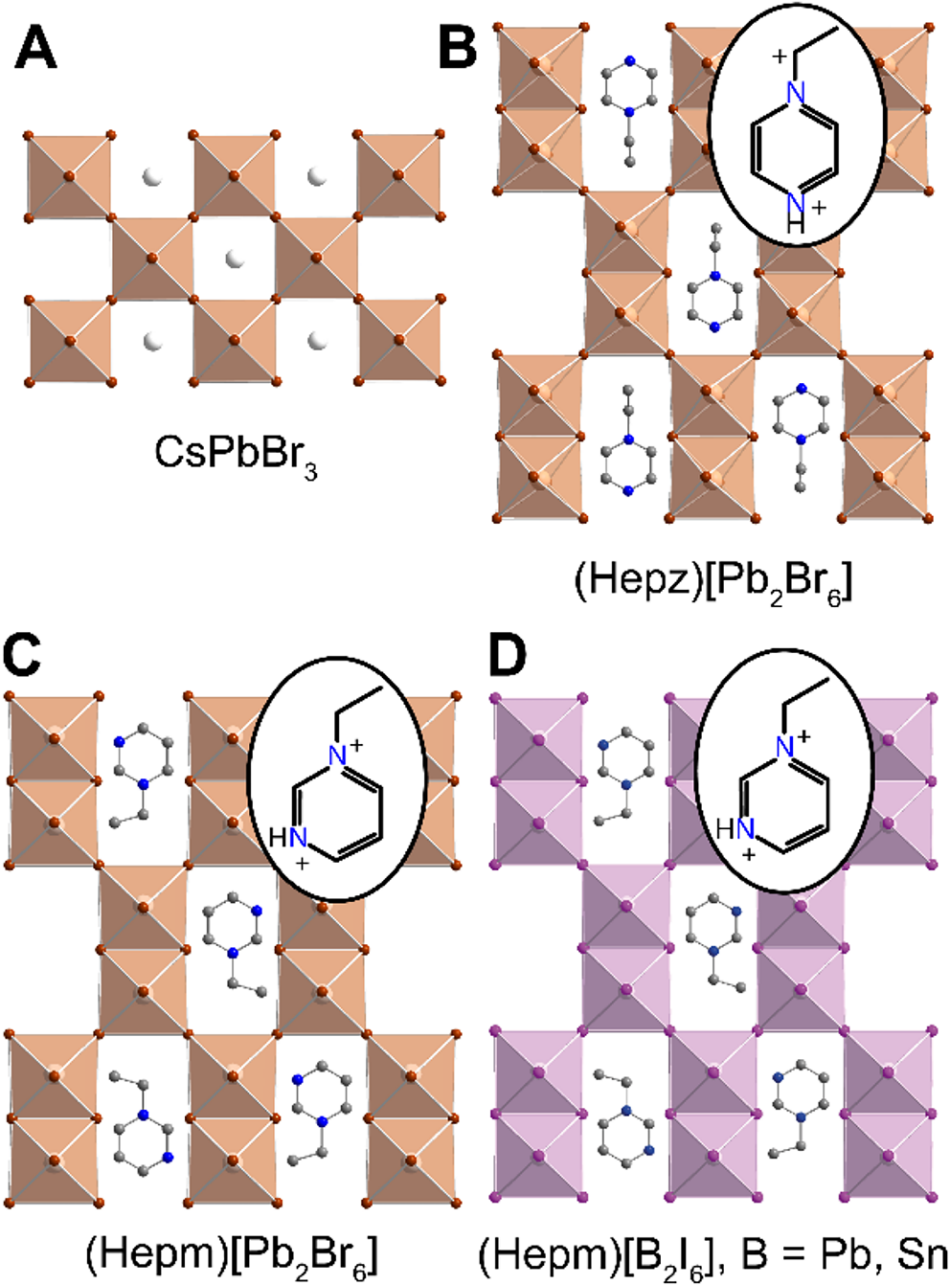
Crystal structures of (A) a 3D halide perovskite, CsPbBr_3_,^30^ and 3D expanded analogs of halide perovskites: (B) (Hepz)­[Pb_2_Br_6_],^25^ and (C) (Hepm)­[Pb_2_Br_6_] and (D) (Hepm)­[Pb_2_I_6_], reported here. The crystal structure of (Hepm)­[Sn_2_I_6_] is represented in Figure S1. Orange and purple polyhedra represent PbBr_6_ and PbI_6_, respectively. White, orange, purple, blue, and gray spheres represent Cs, Br, I, N, and C atoms, respectively. Hydrogen atoms and disordered C/N atoms are omitted for clarity. Hepz^2+^ = 1-ethylpyrazin-1,4-diium, Hepm^2+^ = 1-ethylpyrimidin-1,3-diium.

The main challenge for bulk charge-transfer p-doping is preparing metal-halide semiconductors with a reduced VBM-LUMO energy difference at ambient pressure. Recent work on expanded perovskites at high pressure (>20 GPa) has revealed that the electronic conductivity of expanded perovskites can increase up to 5 orders of magnitude when the VBM-LUMO energy difference is significantly reduced,^31^ demonstrating the potential for bulk charge-transfer doping. However, introducing low-lying LUMO cations inside metal-halide semiconductors poses a significant synthetic challenge. During solution synthesis, side reactions between the metal halide precursors and the redox-active cations are likely to occur, preventing the formation of the desired lattices. Additionally, the solution-state assembly reactions also hinder the preparation of films via solution methods (e.g., spin coating) due to the interaction of polar solvents (i.e., dimethyl sulfoxide, dimethylformamide) with the dications.^26^ Herein, we report on a solid-state alloying method that allows the incorporation of low-lying LUMO cations into expanded perovskite lattices while allowing the preparation of films via inkjet printing. To develop the method, we target the preparation of expanded perovskites containing Hepz^2+^ because the iodide derivatives are expected to display an *E*_g_ of ∼1.2 eV and have not been reported previously. Like (Hepz)­[Pb_2_I_6_], the related iodide expanded lattices containing the (HRpz)^2+^ cation (R = methyl, isopropyl) have not been reported.^26^ We obtain the targeted materials by mechanochemically alloying (Hepz)­[Pb_2_Br_6_] with new expanded perovskites based on the less reactive Hepm^2+^ cation, (Hepm)­[B_2_X_6_] (Figures 1 and S1). Although Hepz^2+^ and Hepm^2+^ are structurally similar, the relative position of the two N in the aromatic ring results in LUMO energies differing by >0.5 eV. Alloying the two Pb-based powders via ball-milling results in mixed A-site perovskites (Hepz)_*x*_(Hepm)_1–*x*_[Pb_2_Br_6_] and (Hepz)_*x*_(Hepm)_1–*x*_[Pb_2_Br_6*x*_I_6–6*x*_], displaying reduced *E*_g_ as low as 1.25(5) eV. Finally, taking advantage of the nanometric nature of ball-milled powders, we prepare films of (Hepm)­[Pb_2_Br_6_] via inkjet printing, providing a proof-of-principle that expanded perovskites are suitable for printed devices. Altogether, this work represents a step forward in developing bulk charge-transfer doping in metal-halide semiconductors.

## 2 Results and Discussion

We obtained μ-sized crystals for (Hepm)­[Pb_2_Br_6_], (Hepm)­[Pb_2_I_6_], and (Hepm)­[Sn_2_I_6_], suitable for single-crystal X-ray diffraction (SCXRD), by combining HX (X = Br, I) solutions of the corresponding metal salt (PbBr_2_, PbI_2_, SnI_2_) and epm­(BF_4_) under air-free conditions. We elucidated the structures of the three new compounds using SCXRD. The space groups of the three new compounds (*Cmmm*) coincide with the (Hepz)­[Pb_2_Br_6_] space group (*Cmmm*).^26^ Overall, the structure of the new expanded lattices (Hepm)­[B_2_X_6_] (B–X = Pb–Br, Pb–I, and Sn–I) are analogous to that of (HRpz)­[Pb_2_Br_6_] (R = methyl, ethyl, isopropyl) and other expanded perovskites (Figures 1C–D and S1 and Tables S1–S3).^26^ The three structures comprise a 3D inorganic sublattice formed by corner-sharing metal-halide octahedra dimers that host Hepm^2+^ in their cavities. Like other (HRpz)­[Pb_2_Br_6_], the angles between the metal-halide dimers are close to 180°, and the organic cations display significant disorder within the cavity. The microcrystalline powders were characterized by powder X-ray diffraction (PXRD) (Figures S4–S6) and infrared spectroscopy (Figure S32). The former confirmed the single-phase nature of the samples.

We calculated the electronic band structures for (Hepz)­[Pb_2_Br_6_] and (Hepm)­[B_2_X_6_] (B–X = Pb–Br, Pb–I, Sn–I) via Density Functional Theory (DFT) based on HSE06 hybrid functional,^32,33^ using the fhi-aims code (Section 4.2.6).^34^ The electronic band structures (Figures 2 and S33–S34) show that the valence band maximum (VBM) consists of B-s and X-p orbitals, like in the related ABX_3_ and other expanded perovskites.^35,36^ In contrast to the APbBr_3_ perovskites, the lowest unoccupied band for (Hepz)­[Pb_2_Br_6_] and (Hepm)­[Pb_2_Br_6_] consists of empty π* orbitals, lying >1.5 eV above the VBM, anticipating an optical gap (*E*_g_) transition between the two levels. Thus, *E*_g_ is mainly related to the energy of Hepz- and Hepm-based bands. The first unoccupied band of Hepz^2+^ is expected to be ∼0.5 eV lower than the Hepm^2+^ band, predicting a narrowed *E*_g_ for (Hepz)­[Pb_2_Br_6_] (Figure 2).

**Figure 2:**
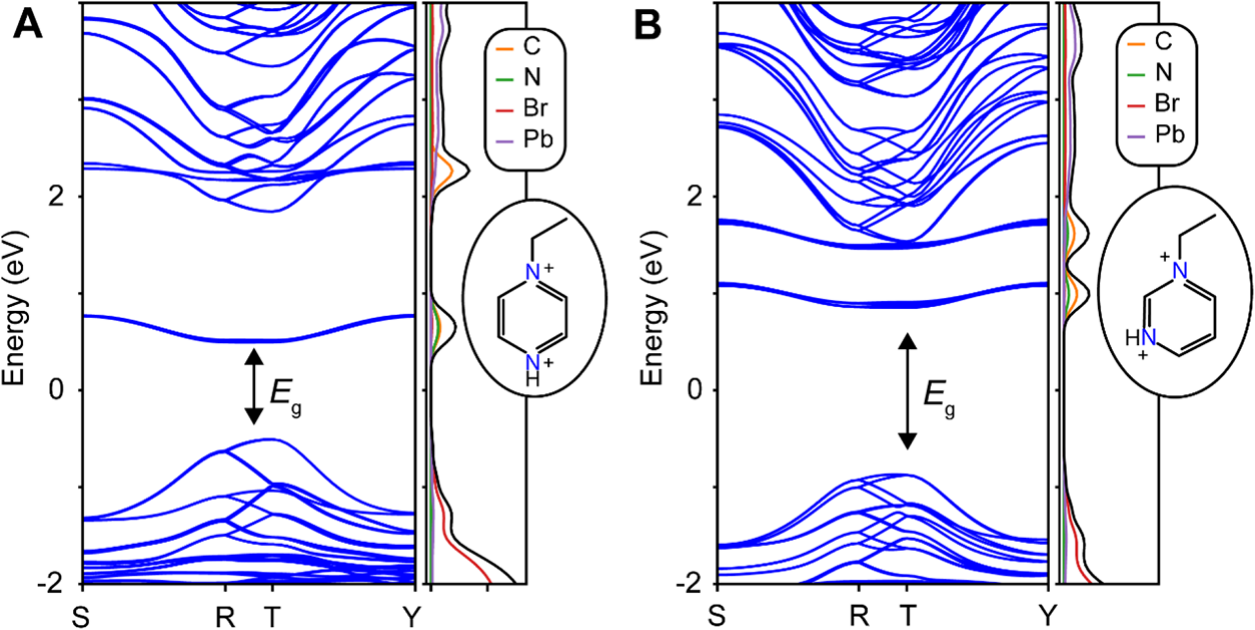
Calculated electronic band structures and density-of-states for (A) (Hepz)­[Pb_2_Br_6_] and (B) (Hepm)­[Pb_2_Br_6_].

To understand the different energies of the Hepz- and Hepm-based bands, we calculated the energy of the molecular orbitals of the Hepz^2+^ and Hepm^2+^ cations (Table S5 and Figure S35). Whereas the energies for the highest occupied molecular orbital (HOMO) of Hepz^2+^ and Hepm^2+^ only differ by less than 0.05 eV, their LUMO energies differ by >0.5 eV. The calculations also reveal that the delocalization of the radical among the six atoms of the aromatic ring causes the low energy of the Hepz^2+^ LUMO. In contrast, the radicals in the Hepm^2+^ LUMO are less delocalized in the ring and, thus, have higher energy (Figure S35). Spin polarization diagrams further confirm that the N topology in Hepz^•+^ results in enhanced charge delocalization (Figure S36). The difference in LUMO energy between Hepz^2+^ and Hepm^2+^ is reminiscent of the reactivity studies in organic chemistry, where the relative position of heteroatoms and substituents in aromatic molecules significantly affects the regioselectivity of aromatic substitution reactions.^37,38^

We then moved to characterize the optical properties of the expanded lattices by transmission (Figures S12–S15) and diffuse reflectance (Figures S16–S19) spectroscopy, extracting the optical gaps (*E*_g_) via Tauc plot analysis (Figures S12–S19). We initially measured the optical gap of (Hepz)­[Pb_2_Br_6_], and our measurements showed that (Hepz)­[Pb_2_Br_6_] displays an *E*_g_ at 1.65(5) eV (Figure 3A), consistent with previously reported *E*_g_ (∼1.7 eV).^26^ We then measured the optical properties of (Hepm)­[B_2_X_6_] (B–X = Pb–Br, Pb–I, Sn–I). The *E*_g_ of (Hepm)­[Pb_2_Br_6_] is 2.30(5) eV (Figure 3B), ∼0.6 eV larger than the *E*_g_ of (Hepz)­[Pb_2_Br_6_]. The increased *E*_g_ again confirms the higher energy of the LUMO of Hepm^2+^ with respect to the Hepz^2+^ LUMO (Figure 3C). The spectra of (Hepm)­[Pb_2_I_6_] and (Hepm)­[Sn_2_I_6_] reveal a narrowed *E*_g_ of 1.60(5) eV and 1.00(5) eV, respectively (Figure 3B). The relativistic contraction of the Pb 6s orbitals^39^ and the low electronegativity of the iodide^36^ explain the reduced *E*_g_ for the Pb–I and Sn–I expanded perovskites (Figure 3C). The calculated electronic band structures of Pb–I and Sn–I expanded perovskites also reflect the reduced *E*_g_ compared to the Pb–Br lattice (Figures S33–S34 and Table S7).

**Figure 3:**
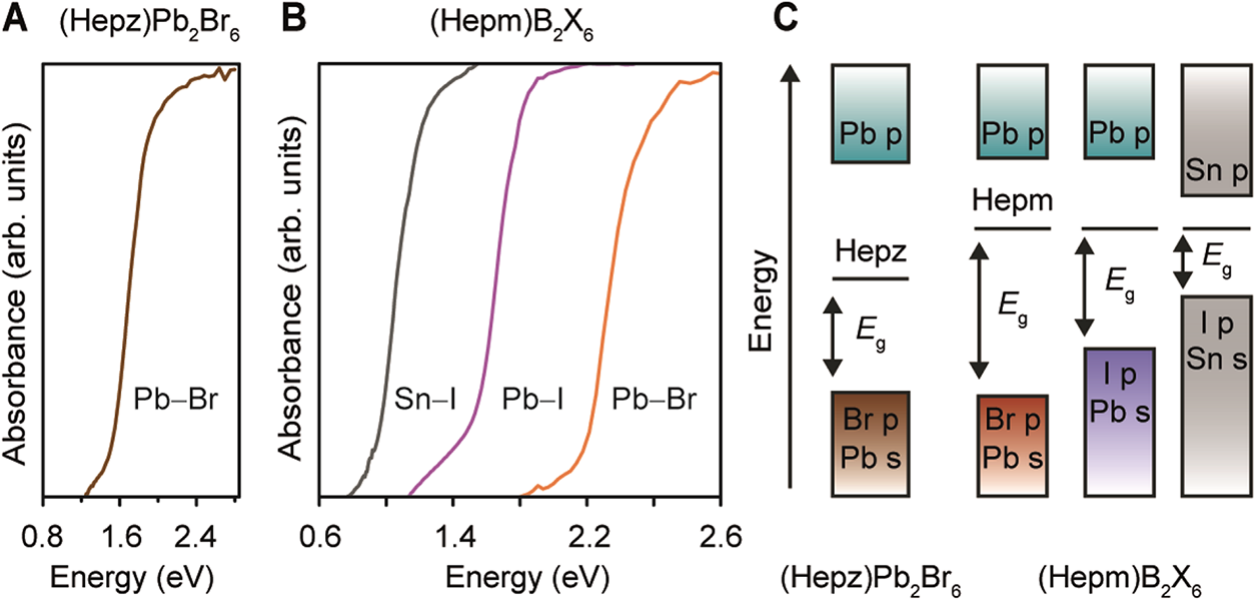
Optical spectra for (A) (Hepz)­[Pb_2_Br_6_] and (B) (Hepm)­[B_2_X_6_] (B–X = Pb–Br, Pb–I, Sn–I), (C) schematic electronic band structures for (Hepz)­[Pb_2_Br_6_] and (Hepm)­[B_2_X_6_] (B–X = Pb–Br, Pb–I, Sn–I), showing the orbital contribution to the band extrema, the Hepz^2+^ and Hepm^2+^ LUMO, and the optical gap (*E*_g_).

We then envisioned that a similar structure could allow preparing expanded lattices with alloyed A-sites, resulting in the incorporation of Hepz^2+^ inside iodide lattices and, thus, narrow *E*_g_. Motivated by the solvent-free mechanochemical alloying of halide perovskites with mixed A-, B-, and X-sites,^40^ we attempted the mechanochemical alloying of our expanded lattices. We ball-milled increasing amounts of (Hepz)­[Pb_2_Br_6_] (0 < *x* < 1) into (Hepm)­[Pb_2_Br_6_] using inert ZrO_2_ balls. The PXRD patterns of the obtained powders show a gradual shift of the diffractions with increasing amounts of *x*, unambiguously confirming the conversion of the precursors into the alloyed (Hepz)_*x*_(Hepm)_1–*x*_[Pb_2_Br_6_] (Figure 4). Thus, the epz-to-epm ratio (*x*) in the initial powder mixture was assumed to be maintained upon ball milling.

**Figure 4:**
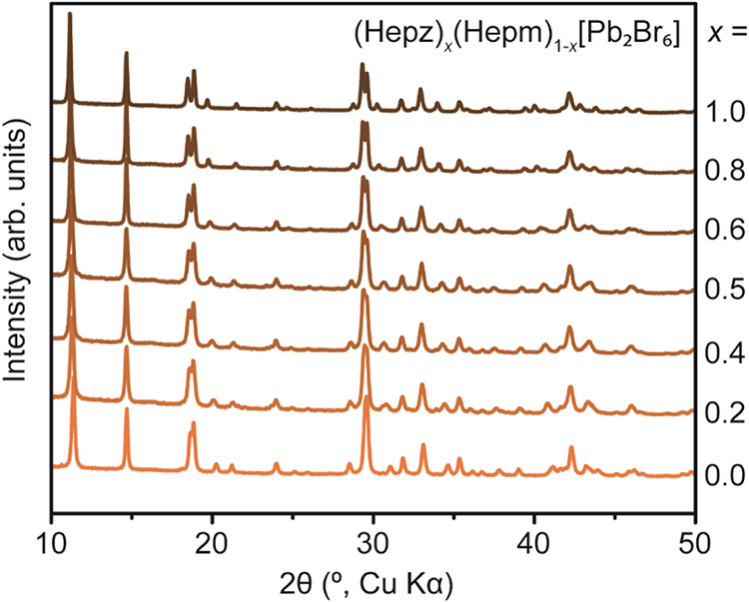
Powder X-ray diffraction (PXRD) patterns for (Hepz)_*x*_(Hepm)_1–*x*_[Pb_2_Br_6_] (0 ≤ *x* ≤ 1).

We then measured the optical properties (Hepz)_*x*_(Hepm)_1–*x*_[Pb_2_Br_6_] by transmission spectroscopy. At *x* percentage as low as 0.01, the spectra of (Hepz)_*x*_(Hepm)_1–*x*_[Pb_2_Br_6_] display an *E*_g_ of 1.80(5) eV, representing a ∼0.5 eV redshift compared to (Hepm)­[Pb_2_Br_6_] (Figure 5A). The same shift is maintained in (Hepz)_*x*_(Hepm)_1–*x*_[Pb_2_Br_6_] with increasing concentrations of Hepz^2+^ (Figure 5C and S20–S23). Thus, the optical gap of (Hepz)_*x*_(Hepm)_1–*x*_[Pb_2_Br_6_] (*x* ≥ 0.01) is dominated by a transition from the VBM to the Hepz-based band, confirming the incorporation of the cation into the lattice.

**Figure 5:**
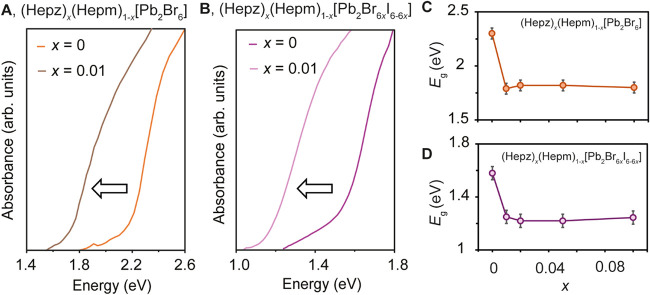
Optical spectra for (A) (Hepz)_*x*_(Hepm)_1–*x*_[Pb_2_Br_6_] (*x* = 0, orange; *x* = 0.01, brown), and (B) (Hepz)_*x*_(Hepm)_1–*x*_[Pb_2_Br_6*x*_I_6–6*x*_] (*x* = 0, purple; *x* = 0.01, pink). Optical gap (*E*_g_) as a function of the percentage of Hepz^2+^ (*x*) in (C) (Hepz)_*x*_(Hepm)_1–*x*_[Pb_2_Br_6_] and (D) (Hepz)_*x*_(Hepm)_1–*x*_[Pb_2_Br_6*x*_I_6–6*x*_].

The slightly smaller shift observed upon alloying (∼0.5 eV) compared to the full *E*_g_ difference between the initial compounds (∼0.6 eV) may be attributed to differences in band dispersion. To estimate the difference, we calculated effective masses at the VBM for (Hepz)­[Pb_2_Br_6_] and (Hepm)­[Pb_2_Br_6_] (Table S6). We obtained that (Hepz)­[Pb_2_Br_6_] exhibits a lower effective mass at the VBM, indicating enhanced band dispersion and explaining the reduced shift in (Hepz)_*x*_(Hepm)_1–*x*_[Pb_2_Br_6_] (*x* ≥ 0.01).

Motivated by the possibility of alloying two different A-sites in expanded perovskites, we attempted alloying (Hepz)­[Pb_2_Br_6_] with (Hepm)­[Pb_2_I_6_] and (Hepm)­[Sn_2_I_6_], respectively. The PXRD data indicate that (Hepz)_*x*_(Hepm)_1–*x*_[Pb_2_Br_6*x*_I_6–6*x*_] is formed upon ball-milling (*x* ≤ 0.2, Figure S7). However, the PXRD patterns for the ball-milled mixture of (Hepz)­[Pb_2_Br_6_] and (Hepm)­[Sn_2_I_6_] display peaks of the two initial phases, indicating that the alloying does not occur (Figure S9). We hypothesized that the unit-cell parameter difference between the Pb–Br and Sn–I lattices may prevent the alloying. To verify the hypothesis, we also attempted the alloying of 20% (Hepm)­[Pb_2_Br_6_] with 80% (Hepm)­[Pb_2_I_6_] (Figure S10) and 80% (Hepm)­[Sn_2_I_6_] (Figure S11), respectively. PXRD data indicate that whereas (Hepm)­[Pb_2_Br_6–6*x*_I_6*x*_] forms (Figure S10), ball-milling (Hepz)­[Pb_2_Br_6_] and (Hepm)­[Sn_2_I_6_] led to a mixture of two phases (Figure S11).

The optical spectra of (Hepz)_*x*_(Hepm)_1–*x*_[Pb_2_Br_6*x*_I_6–6*x*_] (0.01 ≥ *x* ≥ 0.10) reveal an *E*_g_ of 1.25(5), representing a redshift of ∼0.4 eV from (Hepm)­[Pb_2_I_6_] (Figures 5D and S24–S27). The *E*_g_ redshift in (Hepz)_*x*_(Hepm)_1–*x*_[Pb_2_Br_6*x*_I_6–6*x*_] further confirms the incorporation of Hepz^2+^ into the lattice, representing the first example of a Pb–I expanded lattice containing cations with a low-lying LUMO such as Hepz^2+^. To quantify the influence of small percentages of Br in the lattice, we also mechanochemically alloyed 1–10% (Hepm)­[Pb_2_Br_6_] with (Hepm)­[Pb_2_I_6_] (Figures S28–S30). The *E*_g_ of (Hepm)­[Pb_2_Br_6*x*_I_6–6*x*_] at *x* = 0.01–0.10 (1.60(5) eV) was similar to that of (Hepm)­[Pb_2_I_6_] (1.65(5) eV). Thus, small percentages of Br in (Hepz)_*x*_(Hepm)_1–*x*_[Pb_2_Br_6*x*_I_6–6*x*_] or (Hepm)­[Pb_2_Br_6*x*_I_6–6*x*_] do not generate significant blueshifts in the optical gap.

Finally, we investigated solving an additional limitation of expanded analogs of halide perovskites. Films of expanded perovskites containing HRpz^2+^ have not been prepared due to the incompatibility of the cations with highly polar solvents (e.g., dimethyl sulfoxide, dimethylformamide).^26^ With the ball-milled powders at Hepz^2+^- and Hepm^2+^-containing lattices in hand, we sought to provide a proof of principle for the preparation of films via inkjet printing, which allows the preparation of films of nanoparticles suspended in apolar solvents.^41–44^

We suspended ball-milled powders of (Hepm)­[Pb_2_Br_6_] in a high-boiling point solvent (dodecane) with a low-boiling point solvent (hexane) in a 3:1 ratio, respectively, to achieve a final concentration of ∼1 mg mL^–1^. As previously reported with lead-based perovskites,^44,45^ this formulation ensured stable droplet wettability during inkjet printing and controlled droplet ejection and evaporation during the printing process (Table S10) due to the optimal figure of merit (*Z*), which is maintained stable among the working velocity at a value of ∼11 (Table S9 and Figure S38). We then printed (Hepm)­[Pb_2_Br_6_] films of 1 cm^2^ on a glass substrate (Figure 6) using an ink ejection frequency of 5.0 kHz and a resolution of 850 drops per inch. The resulting films exhibited uniformity and minimal surface irregularities, as confirmed by top-view scanning electron microscopy (Figure 6B). We also characterized the optical properties of the films by diffuse reflectance (Figure S31). The inkjet printed films of (Hepm)­[Pb_2_Br_6_] display an optical gap of 2.35(5) eV, which coincides with the optical gap of the bulk sample. To our knowledge, this represents the first example of an expanded perovskite prepared via inkjet printing, which will broaden the applicability of expanded perovskites and allow the fabrication using roll-to-roll printing technology.

**Figure 6:**
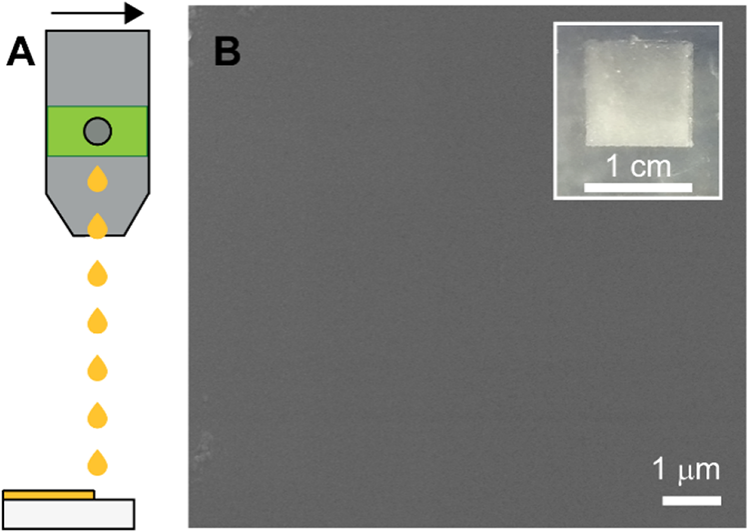
(A) Scheme of an inkjet-printing nozzle used to print films of (Hepm)­[Pb_2_Br_6_]. (B) Scanning electron microscopy image and optical image (inset) of inkjet-printed film of (Hepm)­[Pb_2_Br_6_].

## 3 Conclusions

We show a mechanochemical alloying method that can introduce multiple molecular dopants into expanded halide perovskites and is compatible with inkjet printing. To design the expanded lattices, we exploit the concept of topology in organic chemistry, where the relative position of heteroatoms and substituents in aromatic molecules results in distinct reactivity.^37,38^ Thus, we use two molecular dopants with different LUMO energy but similar structures (Hepz^2+^ and Hepm^2+^) to prepare expanded perovskites. The analogous structure of the two lattices allows the alloy of the two perovskites via solid-state mechanochemistry, displaying a wide range of optical gaps (1.25(5)–2.30(5) eV). It is worth noting that previous attempts based on solution synthesis could not be used to prepare the lattices with the narrowest optical gap, most likely due to side redox reactions between precursors during solution synthesis. By using the mechanochemical method reported here, expanded perovskites with a LUMO-VBM energy difference below 0.5 eV are expected to show significant changes in electrical conductivity. We finally show that ball-milled powders can be used to prepare films via inkjet printing. This method allows the preparation of films of our expanded lattices because they do not require polar solvents incompatible with molecular dopants. Inkjet-printed films exhibit uniformity and minimal surface irregularities, facilitating the future incorporation of expanded perovskites into devices. Altogether, this work represents a significant advance in developing bulk charge-transfer doping in metal-halide semiconductors.

## 4 Experimental Section

### 4.1 Materials

#### 4.1.1 General Considerations

Unless explicitly indicated, all manipulations were conducted using Schlenk-line techniques under an N_2_ atmosphere or in an N_2_-filled glovebox. Toluene and hexane were dried in a solvent purification system. H_3_PO_2_ (50 wt % in H_2_O) was added to concentrated HI (57 wt % in H_2_O, <0.9% H_3_PO_2_) in a 1/100 ratio. Acid solutions (HBr, HI) were bubbled with N_2_ for 15 min before use. We prepared (Hepz)­[Pb_2_Br_6_][Bibr ref26] and 1-ethylpirimidin-1-ium tetrafluoroborate^46^ following the described procedures and stored them in a glovebox freezer. All other reagents were purchased from commercial vendors. A stoichiometric excess of Pb-to-epm (>2) was used in the synthesis of expanded perovskites to avoid the formation of 2D perovskites.^29^

#### 4.1.2 Synthesis of (Hepm)­[Pb_2_Br_6_]

In a Schlenk flask under N_2_, PbBr_2_ (183.5 mg, 0.50 mmol) and epm­(BF_4_) (35 mg, 0.18 mmol) were dissolved in HBr aq. (48% in water, 1 mL). The solution was left in the fridge overnight, and orange crystals, some suitable for single X-ray diffraction, appeared. Crystals were filtered with a fritted glass and dried under vacuum overnight (150 mg, 0.15 mmol, 83% yield). *Anal. Calc*. for C_6_H_10_Br_6_N_2_Pb_2_ C, 7.18%; H, 0.99%; N, 2.79%. *Found:* C, 7.17%; H, 1.17%; N, 3.07%. FTIR (KBr pellet): 3331 (secondary −NH str.), 3027 (aromatic −CH str.), 2964, 2917, 2850, 1385, 726 (aliphatic −CH str.), 1600, 1656 (−C=N str.), 1609, 1581, 1479 (−C=C str.), 694, 669 (−C=C bend.) cm^–1^.

#### 4.1.3 Synthesis of (Hepm)­[Pb_2_I_6_]

In a Schlenk flask under N_2_, PbI_2_ (922 mg, 2.00 mmol) and epm­(BF_4_) (65 mg, 0.33 mmol) were dissolved in a HI solution (57% in water, 1.8 mL), which was previously degassed with N_2_. The solution was stirred at 80 °C for 15 min, and black crystals, some suitable for single X-ray diffraction, appeared. Crystals were filtered with a fritted glass, washed with N_2_-bubbled acetonitrile (2 mL) and toluene (50 mL), and dried overnight under vacuum (200 mg, 0.16 mmol, 48% yield).

#### 4.1.4 Synthesis of (Hepm)­[Sn_2_I_6_]

In a Schlenk flask under N_2_, SnI_2_ (745 mg, 2.00 mmol) and epm­(BF_4_) (60 mg, 0.30 mmol) were dissolved in a HI solution (57% in water, 1.2 mL), which was previously degassed with N_2_. The solution was stirred at 60 °C for 20 min. The resulting black crystals, some suitable for single X-ray diffraction, were filtered with a fritted glass and washed twice with N_2_-bubbled toluene (50 mL). Crystals were dried overnight under a vacuum (250 mg, 0.23 mmol, 75% yield).

#### 4.1.5 Mechanochemical Synthesis of (Hepz)_*x*_(Hepm)_1–*x*_[Pb_2_Br_6_]

Powders of (Hepm)­[Pb_2_Br_6_] (27.0–29.7 mg, 0.027–0.030 mmol) and (Hepz)­[Pb_2_Br_6_] (0.3–3.0 mg, 0.3–3.0 μmols) were added in different proportions (*x* = 0.01–0.8) in a 2 mL polypropylene Eppendorf vial. Two 3 mm-diameter ZrO_2_ balls were added to the mixture, which was ball-milled for 20 min at 30 Hz in a Retsch MM400 miller. The product was stored in a N_2_-filled glovebox.

#### 4.1.6 Mechanochemical Synthesis of (Hepz)_*x*_(Hepm)_1–*x*_[Pb_2_Br_6*x*_I_6–6*x*_]

Powders of (Hepm)­[Pb_2_I_6_] (27.0–29.7 mg, 0.021–0.023 mmol) and (Hepz)­[Pb_2_Br_6_] (0.3–3.0 mg, 0.3–3.0 μmols) were added in different proportions (*x* = 0.01–0.20) in a 2 mL polypropylene Eppendorf vial. Two 3 mm-diameter ZrO_2_ balls were added to the mixture, which was ball-milled for 20 min at 30 Hz in a Retsch MM400 miller. The product was stored in a N_2_-filled glovebox.

#### 4.1.7 Mechanochemical Synthesis of (Hepm)­[Pb_2_Br_6*x*_I_6–6*x*_]

Powders of (Hepm)­[Pb_2_I_6_] (27.0–29.7 mg, 0.021–0.023 mmol) and (Hepm)­[Pb_2_Br_6_] (0.3–3.0 mg, 0.3–3.0 μmols) were added in different proportions (*x* = 0.01–0.10) in a 2 mL polypropylene Eppendorf vial. Two 3 mm-diameter ZrO_2_ balls were added to the mixture, which was ball-milled for 20 min at 30 Hz in a Retsch MM400 miller. The product was stored in a N_2_-filled glovebox.

### 4.2 Methods

#### 4.2.1 Single-Crystal X-ray Diffraction

Data for (Hepm)­[Pb_2_Br_6_], (Hepm)­[Pb_2_I_6_], and (Hepm)­[Sn_2_I_6_] were collected at Bruker D8 Venture at the research facilities of the *Universitat de Barcelona* (CCiTUB) at Mo Kα (λ = 0.71073). The crystals were mounted with Paratone N grease on a MicroMounts loop and placed in the N_2_ stream of an Oxford Cryosystem. Frames were collected, and unit-cell parameters were refined against all data. The crystals did not show significant decay during data collection. Space-group assignments were based upon systematic absences, E-statistics, agreement factors for equivalent reflections, and successful refinement of the structures. Structures were solved using the intrinsic phasing method implemented in SHELXT-2014.^47^ Solutions were refined against all data using the SHELXL-2018/34 ^48^ software package and OLEX.^49^ The refining includes anisotropic displacement parameters except for the hydrogen atoms, which were calculated. Details regarding data quality and a summary of the residual values of the refinements are listed in Tables S1–S3. In the (Hepm)­[Pb_2_Br_6_] and (Hepm)­[Sn_2_I_6_] structures, the organic cation is disordered in 4 positions with a 0.25 ratio (Figures S2–S3). The positions have a *C*2/*m* symmetry. In the (Hepm)­[Pb_2_I_6_] structure, the organic cation is disordered in 8 positions with a 0.125 ratio. The positions have a *C*2/*m*/*m* symmetry.

#### 4.2.2 Powder X-ray Diffraction

Powder X-ray diffraction (PXRD) measurements were performed at the research facilities of the *Universitat de Barcelona* (CCiTUB) on a PANanalytical *X’Pert PRO MPD* θ/θ, equipped with a Cu anode and a PIXcel detector with an active length of 3.347°. We performed 2θ/θ scans from 4 to 50° with a step size of 0.0263° and a measuring time of 200 s per step. For (Hepz)_*x*_(Hepm)_1–*x*_[Pb_2_Br_6_] (*x* = 0–1), the measurements were performed in transmission geometry with powders sandwiched between Mylar films. Sandwiches were prepared inside a glovebox. This configuration did not provide a tightly sealed environment, and thus, it could not be used for (Hepz)_*x*_(Hepm)_1–*x*_[Pb_2_Br_6*x*_I_6–6*x*_] (*x* = 0.01–0.2). For (Hepz)_*x*_(Hepm)_1–*x*_[Pb_2_Br_6*x*_I_6–6*x*_], we measured PXRD in transmission geometry, and the powders were sandwiched between Kapton films inside a N_2_-filled glovebox. Finally, we performed PXRD measurements for (Hepm)­[B_2_X_6_] (B–X = Pb–I, Sn–I) using 0.5 mm of diameter Lindemann glass capillaries prepared inside of an N_2_ glovebox. Sequential measurements did not show sample degradation during the measurement’s time scale. Simulated PXRD patterns were calculated using Mercury software and the crystallographic information files (CIFs) from SCXRD structures.

#### 4.2.3 Optical Measurements

##### 4.2.3.1 Sample Preparation

Inside an N_2_-filled glovebox, an ink was prepared by ball-milling 20 mg of the expanded perovskite with hexane (0.2 mL) in a 2 mL polypropylene Eppendorf containing two ZrO_2_ balls (3 mm diameter). The Eppendorf was sealed using grease and parafilm. The powders were ball milled at room temperature for 15 min at 30 Hz in a Retsch MM400 miller. After that time, hexane was added (0.5 mL), and the suspension was sonicated for 20 min. Inside a N_2_-filled glovebox, the resulting ink (200 μL) was drop-casted on top of a glass slide. Once dried, the powders were encapsulated by drop-casting (100 μL × 3) a PMMA solution in toluene (10 mg mL^–1^) on top of the powders.

##### 4.2.3.2 Transmission and Diffuse Reflectance Measurements

Transmission and diffuse reflectance measurements were acquired by illuminating the sample with a 3 mm × 3 mm spot size with monochromatic light from a Xe and quartz halogen dual lamp source with a power density of 10–40 μW cm^–2^ and measuring the transmitted or reflected light with an InGaAs photodetector, thanks to an integrating sphere (Bentham PV300 EQE system). For (Hepz)­[Pb_2_Br_6_], (Hepm)­[Pb_2_Br_6_], (Hepm)­[Pb_2_I_6_], and (Hepm)­[Sn_2_I_6_], transmission and diffuse reflectance data were acquired. The optical gap (*E*_g_) error was estimated as the standard deviation of the two values. For (Hepz)_*x*_(Hepm)_1–*x*_[Pb_2_Br_6_] and (Hepz)_*x*_(Hepm)_1–*x*_[Pb_2_Br_6*x*_I_6–6*x*_] only transmission data were acquired. Measurements were performed in an open atmosphere. We performed sequential measurements to ensure that samples did not evolve during the measurements. Samples encapsulated with PMMA did not show any evolution in the time scale of the measurements.

##### 4.2.3.3 Transmission and Diffuse Reflectance Data Treatment

The transmission data was transformed to absorbance using the formula *A* = 2 – log­(%*T*). The diffuse reflectance data were transformed using the Kubelka–Munk function,^50^ which was used to calculate α/*S* from the reflectance data, where α is the pseudoabsorption coefficient, and *S* is the scattering coefficient.

Following procedures by Poeppelmeier et al.,^51^ Tauc plots were constructed representing *A* ^2^ (for transmission data) or (α*S* ^–1^)^2^ (diffuse reflectance data) against the photon energy and extrapolating *E*_g_ from *A* ^2^ = 0 and (α*S* ^–1^)^2^ = 0. For completeness,^52^ we also extracted *E*_g_ by extrapolating Tauc plots as (*A*·*h*υ)^2^ = 0 and (α*S* ^–1^·*h*υ)^2^ = 0. Both Tauc Plots lead to statistically similar results (Table S4).

#### 4.2.4 Elemental Analysis

Elemental analysis was performed in an EA3100 Eurovector in configuration NCH. The combustion was performed at an oven temperature of 980 °C and the temperature of the chromatographic column of 90 °C. The equipment had a carrier helium pressure of 90 kPa or 120 mL min^–1,^ while the reference helium pressure was 20 kPa. The oxygen pressure was at 161 kPa. To facilitate the combustion of the sample, V_2_O_5_ was added. For the linear calibration of the results, sulphanilamide is used as a pattern. Elemental analyses for (Hepm)­[Pb_2_I_6_] and (Hepm)­[Sn_2_I_6_] were not performed due to their high hygroscopicity.

#### 4.2.5 Infrared Spectroscopy

Inside a glovebox, 5 mg of each sample was mixed with 100 mg of dried KBr, used as a dispersing agent, and placed in a press at 10 tons under an N_2_ atmosphere for 5 min to create pellets for the infrared spectroscopy measurements. Transmission infrared spectroscopy was performed on air using a Nicolet 5700 FT-IR spectrophotometer with a step size of 1.9285 cm^–1^, spanning the range from 400 to 4000 cm^–1^. The spectrum was visualized using the OMNIC software, where the bands were assigned.^53^ Infrared spectroscopy for (Hepm)­[Pb_2_I_6_] and (Hepm)­[Sn_2_I_6_] was not performed due to their high hygroscopicity.

#### 4.2.6 Computational Methods

A theoretical DFT study of the systems’ electronic structure was performed using fhi-aims code^32,33^ and the PBE exchange-correlation functional,^54^ including dispersion effects by a many-body approach^55^ for the structural optimization of the unit cells. The band structure for the optimized structures was performed using the hybrid HSE06 functional, which included scalar relativistic ZORA and spin–orbit corrections.^34^ All the calculations were carried out using a tight quality basis set^32^ and 16 *k*-points, respectively. The effective masses (Table S6) were calculated using the fhi-aims data with the postprocessing effmass code employing the finite difference approach.^56^

#### 4.2.7 Inkjet Printing

##### 4.2.7.1 Ink Preparation

Powders of (Hepm)­[Pb_2_Br_6_] (100 mg) were mixed with hexane (0.2 mL) in a 2 mL polypropylene Eppendorf containing two 3 mm-diameter ZrO_2_ balls. The mixture was ball-milled at room temperature for 2 h at 30 Hz. The 3 mm-diameter ZrO_2_ balls were then replaced with 15 1 mm-diameter ZrO_2_ balls, and the mixture was ball-milled for 6 h at 30 Hz in a Retsch MM400 miller. After the milling, hexane (0.5 mL) was added, and the suspension was sonicated for 20 min. The balls were separated from the solution using a 1 mm mesh, and the solvent was evaporated at room temperature. The ink was prepared by mixing a high-boiling point solvent (dodecane) with a low-boiling point solvent (hexane) in a 3:1 ratio to achieve a final concentration of approximately 1 mg mL^–1^. This formulation ensures stable droplet jettability during inkjet printing.^44,57–59^

##### 4.2.7.2 Printing Process

The inks were printed using an ink ejection frequency of 5.0 kHz and a resolution of 850 drops per inch (DPI). The inks, filtered and adjusted to a viscosity of ∼2.2 cP, were printed with a fixed drop volume of 10 pL through a 21 μm diameter nozzle using a Fujifilm Dimatix cartridge (Figure S37). Glass was used as a substrate. During the (Hepm)­[Pb_2_Br_6_] layer deposition, the platen temperature was maintained at room temperature (20 °C) under inert glovebox conditions (H_2_O and O_2_ levels <1 ppm) to ensure proper droplet spreading and adhesion throughout the layered stack. The postprinting curing process involved hot vacuum annealing at 130 °C for 15 min. For reliable inkjet printing, the size of dispersed nanomaterials must be smaller than 1/50 of the nozzle diameter (δ) to prevent clogging.^60^ Drop formation is governed by rheological parameters, including density (ρ), viscosity (η), and surface tension (γ), which reflect inertial, viscous, and capillary forces, respectively. These parameters define the dimensionless figure of merit (*Z*), which is inversely proportional to the Ohnesorge number (*Oh*) and independent of fluid velocity.^57^

The ink’s measured surface tension, viscosity, and density were 26 dyn cm^–1^, 0.021 g cm^–1^ s^–1^, and 1.08 g cm^–3^, respectively (Table S8). Based on the rheological study (Table S9), the calculated *Z* value for the (Hepm)­[Pb_2_Br_6_] ink is 11.05, which lies within the optimal printing range (1 < *Z* < 14) that predicts stable droplet formation.^61^ (Figure S38). These parameters are critical for forming uniform, low-roughness (Hepm)­[Pb_2_Br_6_] thin films and for avoiding the “coffee ring” effect caused by preferential evaporation at the contact line.^62^ This effect can be mitigated by inducing inward Marangoni flow,^63^ enabled here by combining high- and low-boiling point solvents, which control droplet evaporation during printing. Other quality determinants, such as printing resolution (minimum stripe spacing), layer homogeneity (pinhole-free surfaces), and adhesion (contact angle pinning), were optimized by adjusting the frequency of drop injection, nozzle and substrate temperature, and number of drops per inch. Inkjet printing conditions used for achieving stable and reproducible jettability are summarized in Table S10.

## Supplementary Material



## Notes

Additional experimental data, including crystallographic tables, powder X-ray diffraction patterns, optical measurements, infrared spectrum, calculated electronic structures, and inkjet printing optimization parameters (PDF)

## Abbreviations

Hepz^2+^: 1-ethylpyrazin-1,4-diium
Hepm^2+^: 1-ethylpyrimidin-1,3-diium
