# Supplementary material for: Mechanochemical Alloying of Molecular Dopants in Expanded Analogs of Halide Perovskites
Source: Inorg Chem. 2025 Aug 20;64(34):17279–87. doi: 10.1021/acs.inorgchem.5c02436 (PMC12406187; doi:10.1021/acs.inorgchem.5c02436)
Supplement: Supplementary file 1 [file ic5c02436_si_001.pdf]

Supporting information

Mechanochemical Alloying of Molecular Dopants in Expanded Analogs of Halide Perovskites

Xavier Rodriguez-Rubio,[1] Giovanni Vescio,[2-3] Joshua D. Forero,[2] Albert Cirera,[2] Horst Puschmann,[4] Eliseo Ruiz,[1] Sergi Hernández,[2] Roc Matheu[1*]

[1] Department of Inorganic and Organic Chemistry, Institut de Química Teòrica i Computacional, Universitat de Barcelona, Carrer Martí i Franquès 1, 08028 Barcelona, Spain

[2] Department of Electronic and Biomedical Engineering, Institute of Nanoscience and Nanotechnology, Universitat de Barcelona, Carrer Martí i Franquès 1, 08028 Barcelona, Spain

[3] Department of Electronic Engineering, Universitat Politècnica de Catalunya, Carrer Jordi Girona, 1, Edifici C4, 08034 Barcelona, Spain.

[4] Department of Chemistry, University of Durham, South Road, Durham DH1 3LE, UK

* Correspondence to roc.matheu@ub.edu

Table S1. Crystallographic data for (Hepm)[Pb_2Br_6]

**Compound**	**(Hepm)[Pb_2Br_6]**
Empirical formula	$C_6N_2H_{10}Pb_2Br_6$
Formula weight (g mol^{-1})	1004.015
Temperature (K)	300
Crystal system	Orthorhombic
Space group	*Cmmm*
a, *b*, *c* (Å)	8.8117(18)<br>16.674(4)<br>6.0206(14)
α, β, γ (°)	90<br>90<br>90
Volume (Å^3)	884.6(3)
Z	2
Density, calc. (g cm^{-3})	3.792
Absorption coeff. (mm^{-1})	32.617
F(000)	855.6
Crystal size (mm^3)	0.04 × 0.04 × 0.03
Radiation	MoKα (λ = 0.71073)
2θ range (°)	4.88, 55
Index ranges	$-11 \leq h \leq 9$<br>$-21 \leq k \leq 21$<br>$-7 \leq l \leq 7$
Reflections collected/unique	4326/614
Completeness to θ_{max}	99.4
Data/restraints/parameters	614/125/62
Goodness-of-fit on F^2	1.044
Final *R* indices [$I > 2\sigma(I)$][a]	$R_1 = 0.0332$
Final *R* indices [all data][a]	$R_1 = 0.0376$<br>$wR_2 = 0.0891$
Largest diff. peak/hole (e Å^{-3})	1.90/-1.01

[a] $R_1 = \Sigma||F_o| - |F_c||/\Sigma|F_o|$, $wR_2 = [\Sigma w(F_o^2 - F_c^2)^2/\Sigma(F_o^2)^2]^{1/2}$

Table S2. Crystallographic data for $(Hepm)[Pb_2I_6]$

**Compound**	**$(Hepm)[Pb_2I_6]$**
Empirical formula	$C_6N_2H_{10}Pb_2I_6$
Formula weight (g mol^{-1})	1286.020
Temperature (K)	300
Crystal system	Orthorhombic
Space group	*Cmmm*
a, *b*, *c* (Å)	9.1903(3)<br>17.6056(6)<br>6.4069(2)
α, β, γ (°)	90<br>90<br>90
Volume ($Å^3$)	1036.64(6)
Z	2
Density, calc. (g cm^{-3})	4.120
Absorption coeff. (mm^{-1})	25.173
F(000)	1069.3
Crystal size (mm^3)	0.04 × 0.04 × 0.03
Radiation	MoKα (λ = 0.71073)
2θ range (°)	4.62, 51.38
Index ranges	$-11 \leq h \leq 11$<br>$-21 \leq k \leq 21$<br>$-7 \leq l \leq 7$
Reflections collected/unique	4529/597
Completeness to θ_{max}	100
Data/restraints/parameters	597/125/74
Goodness-of-fit on F^2	1.064
Final R indices [$I > 2\sigma(I)$][a]	$R_1 = 0.0363$
Final R indices [all data][a]	$R_1 = 0.0390$<br>$wR_2 = 0.0954$
Largest diff. peak/hole (e $Å^{-3}$)	2.50/-2.34

[a] $R_1 = \Sigma||F_o| - |F_c||/\Sigma|F_o|$, $wR_2 = [\Sigma w(F_o^2 - F_c^2)^2/\Sigma(F_o^2)^2]^{1/2}$

Table S3. Crystallographic data for (Hepm)[Sn_2I_6]

**Compound**	**(Hepm)[Sn_2I_6]**
Empirical formula	$C_6N_2H_{10}Sn_2I_6$
Formula weight (g mol^{-1})	1109.007
Temperature (K)	300
Crystal system	Orthorhombic
Space group	*Cmmm*
a, *b*, *c* (Å)	9.1551(5)<br>17.4453(8)<br>6.3365(3)
α, β, γ (°)	90<br>90<br>90
Volume (Å^3)	1012.02(9)
Z	2
Density, calc. (g cm^{-3})	3.639
Absorption coeff. (mm^{-1})	11.608
F(000)	949.1
Crystal size (mm^3)	0.04 × 0.03 × 0.01
Radiation	MoKα (λ = 0.71073)
2θ range (°)	4.66, 52.76
Index ranges	$-11 \leq h \leq 11$
	$-21 \leq k \leq 21$
	$-7 \leq l \leq 6$
Reflections collected/unique	5636/624
Completeness to θ_{max}	99.6
Data/restraints/parameters	624/119/76
Goodness-of-fit on F^2	1.102
Final *R* indices [$I > 2\sigma(I)$][a]	$R_1 = 0.0353$
Final *R* indices [all data][a]	$R_1 = 0.0455$
	$wR_2 = 0.0944$
Largest diff. peak/hole (e Å^{-3})	1.43/-1.17

[a] $R_1 = \Sigma||F_o| - |F_c||/\Sigma|F_o|$, $wR_2 = [\Sigma w(F_o^2 - F_c^2)^2/\Sigma(F_o^2)^2]^{1/2}$

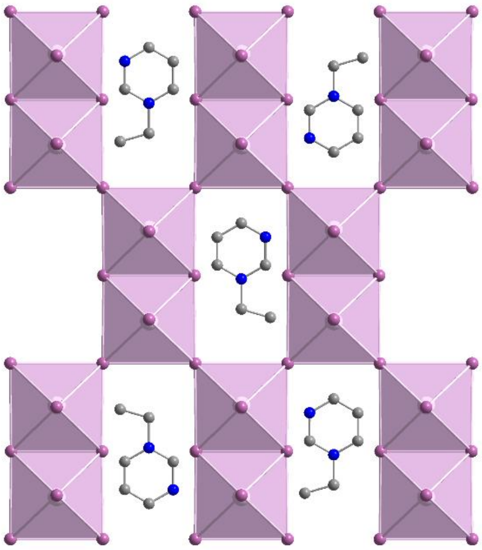

Figure S1. Single-crystal X-ray diffraction (SCXRD) structure for (Hepm)[Sn_2I_6] at 300 K. Purple polyhedra represent SnI_6. Purple, blue, and gray spheres represent I, N, and C atoms, respectively. Hydrogen atoms and disordered C/N atoms are omitted for clarity.

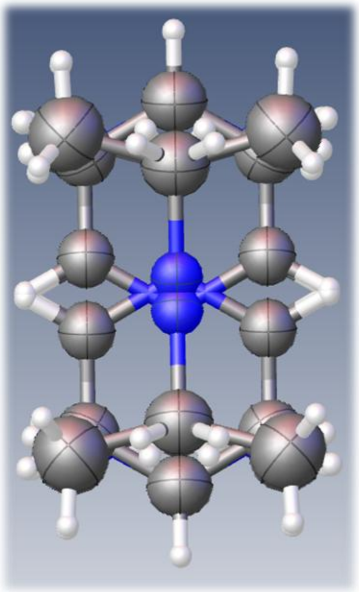

Figure S2. Representation of the overlapping of all symmetry-equivalent organic dications in (Hepm)[Pb_2Br_6], occupying the space formed by corner-sharing metal-halide octahedra dimers.

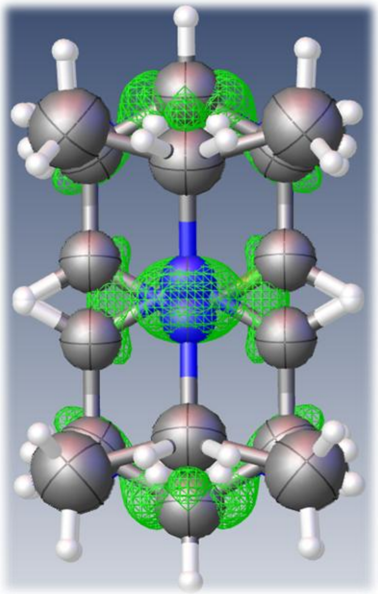

Figure S3. Residual density plot of all symmetry equivalent molecules occupying the space formed by corner-sharing metal-halide octahedra dimers. The residual density is the minimum, given the impossibility of alignment of these equivalents due to the symmetry.

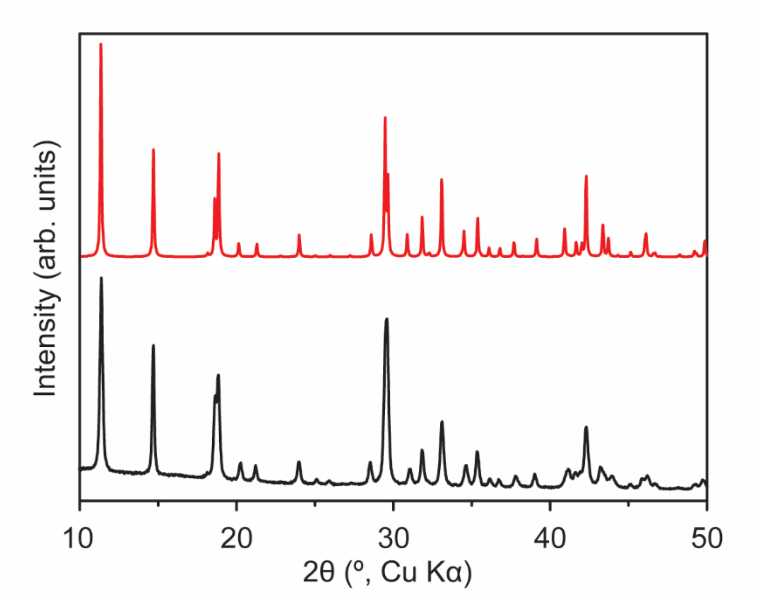

Figure S4. Powder X-ray diffraction (PXRD) pattern for (Hepm)[Pb_2Br_6] (black) and the simulated pattern from the room-temperature SCXRD structure (red).

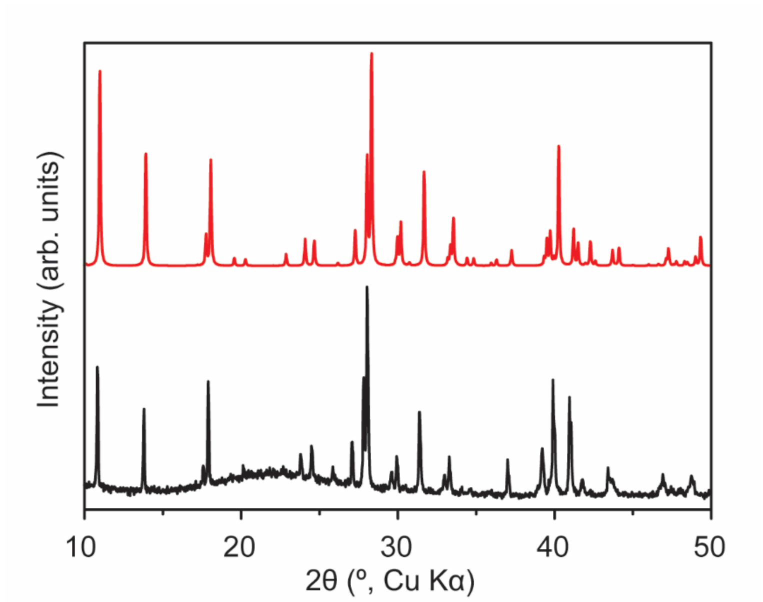

Figure S5. PXRD pattern for (Hepm)[Pb_2I_6] (black) and the simulated pattern from the SCXRD structure at 100 K (red).

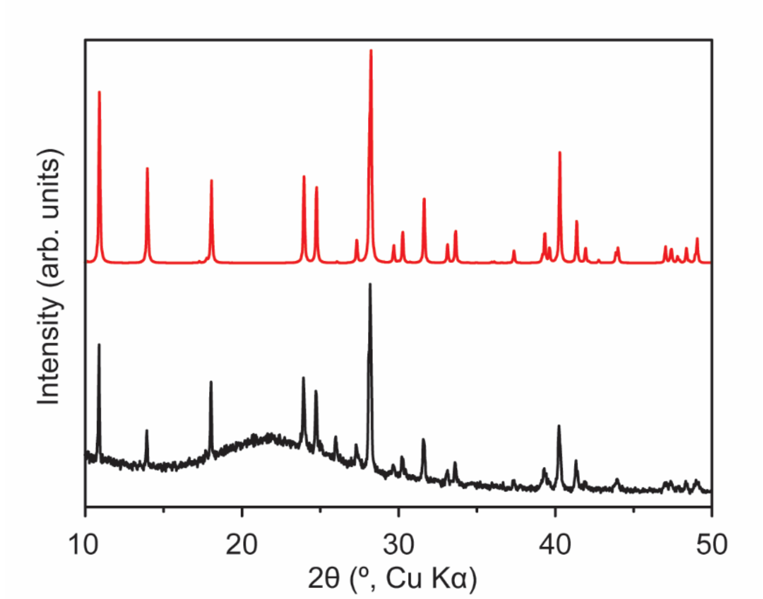

Figure S6. PXRD pattern for (Hepm)[Sn_2I_6] (black) and the simulated pattern from the room-temperature SCXRD structure (red).

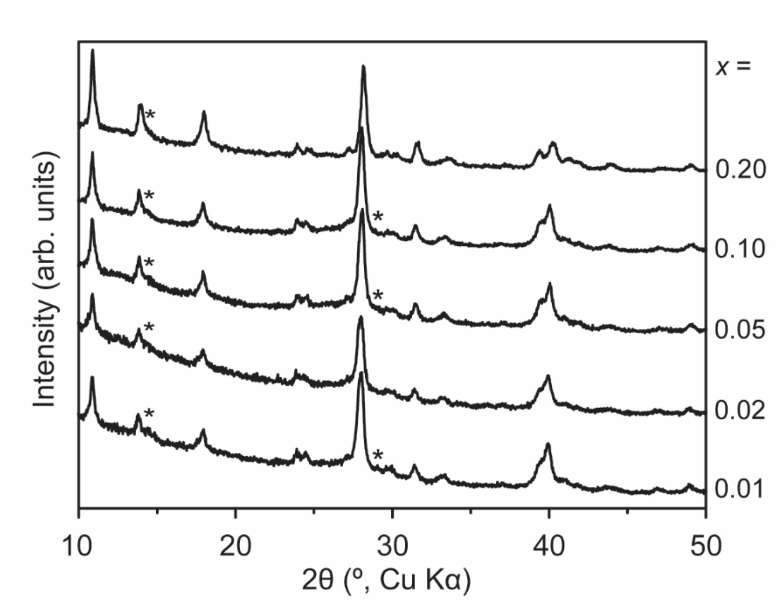

Figure S7. PXRD pattern for $(Hepz)_x(Hepm)_{1-x}[Pb_2Br_{6x}I_{6-6x}]$. Asterisks indicate peaks associated with the Kapton® film (Figure S8).

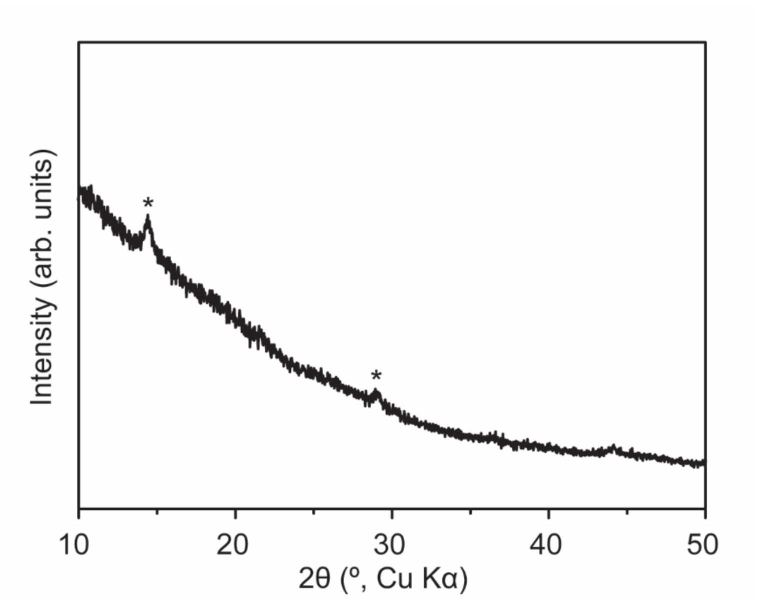

Figure S8. PXRD pattern of a blank Kapton® film.

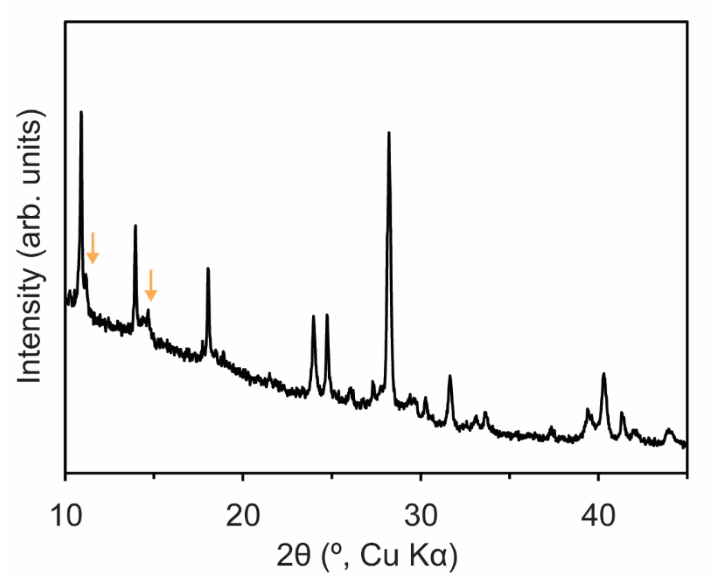

Figure S9. PXRD pattern of a mixture of 10% (Hepz)[Pb_2Br_6] and 90% (Hepm)[Sn_2I_6] that was ball-milled for 20 min at 30 Hz with 3-mm-diameter ZrO_2 balls, displaying peaks of the two initial precursors. Orange arrows indicate peaks related to (Hepz)[Pb_2Br_6]

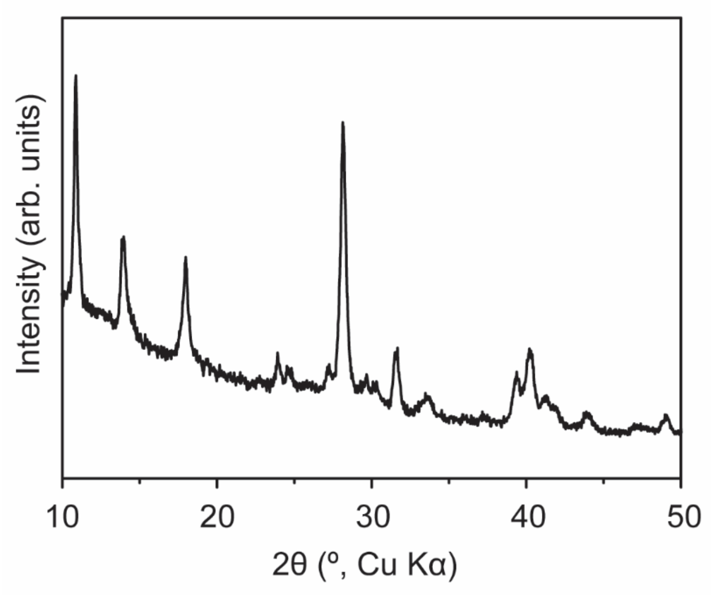

Figure S10. PXRD pattern for (Hepm)[$Pb_2Br_{1.2}$ $I_{5.8}$]

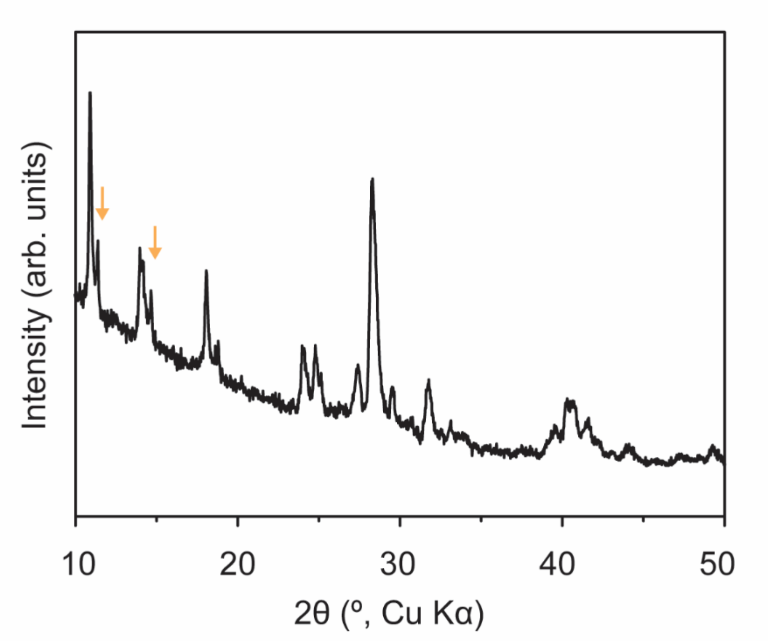

Figure S11. PXRD pattern for a mixture of 20% (Hepm)[Pb_2Br_6] and 80% of (Hepm)[Sn_2I_6] that was ball-milled for 20 min at 30 Hz with 3-mm-diameter ZrO_2 balls, displaying peaks of the two initial precursors. Orange arrows indicate peaks related to (Hepm)[Pb_2Br_6]

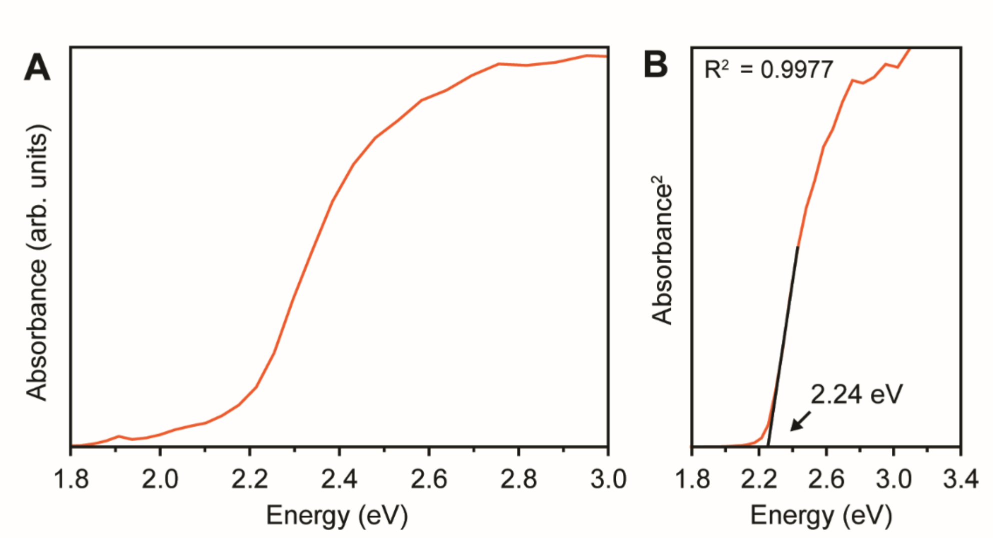

Figure S12. (**A**) Absorbance spectrum for (Hepm)[Pb_2Br_6]. (**B**) Tauc plot for (Hepm)[Pb_2Br_6].

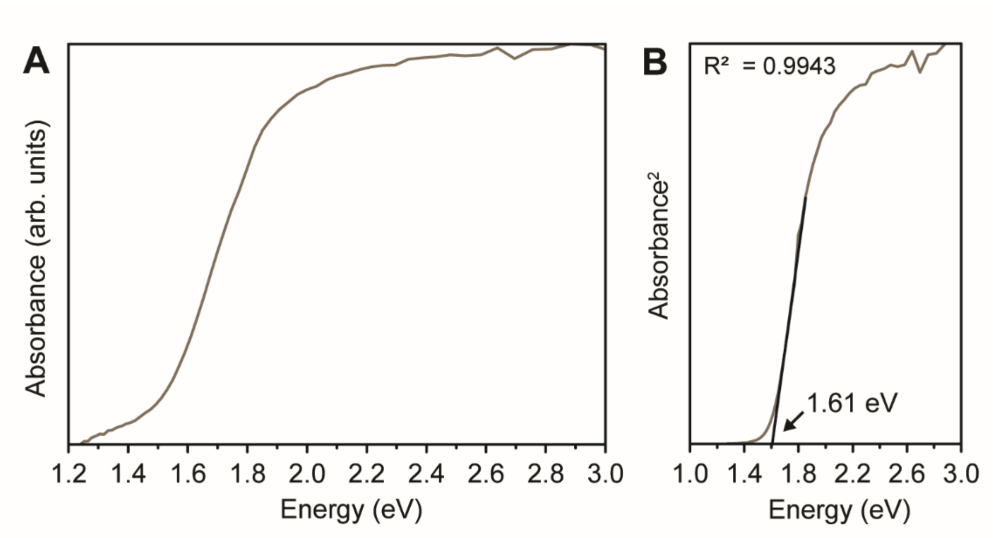

Figure S13. (**A**) Absorbance spectrum for (Hepz)[Pb_2Br_6]. (**B**) Tauc plot for (Hepz)[Pb_2Br_6].

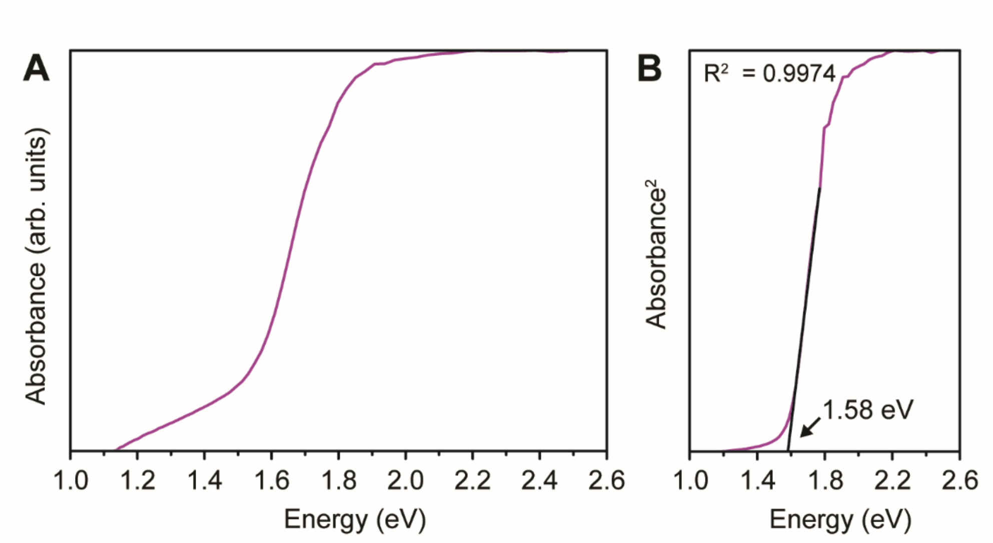

Figure S14. (**A**) Absorbance spectrum for (Hepm)[Pb_2I_6]. (**B**) Tauc plot for (Hepm)[Pb_2I_6].

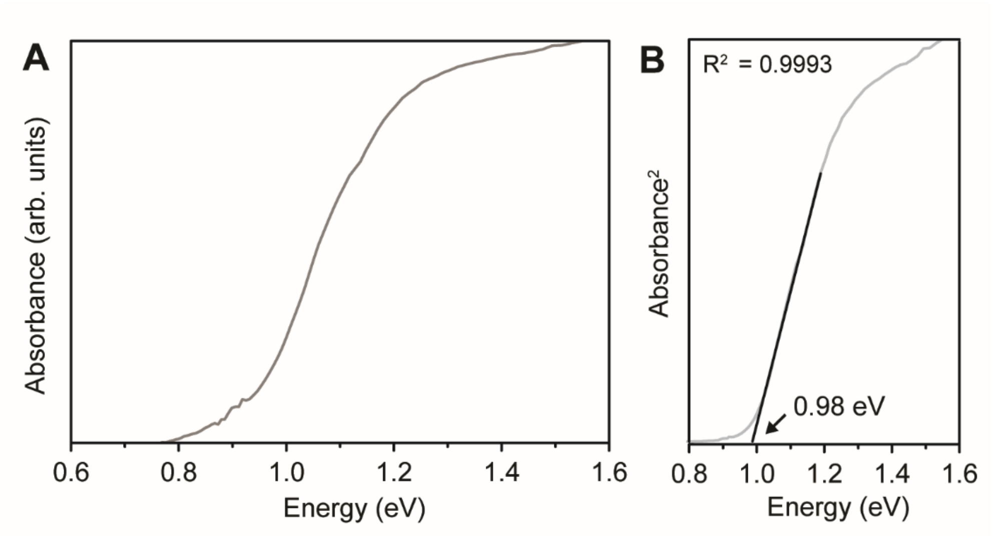

Figure S15. (**A**) Absorbance spectrum for (Hepm)[Sn_2I_6]. (**B**) Tauc plot for (Hepm)[Sn_2I_6].

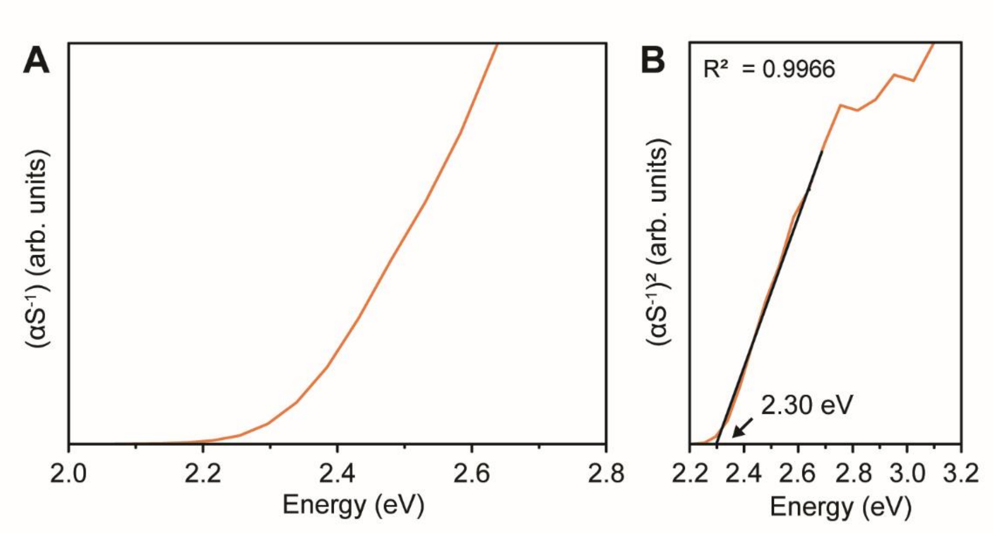

Figure S16. (**A**) Diffuse reflectance spectrum for (Hepm)[Pb_2Br_6]. The diffuse reflectance data were transformed using the Kubelka–Munk function (α and S are the pseudo-absorption and scattering coefficients, respectively). (**B**) Tauc plot for (Hepm)[Pb_2Br_6].

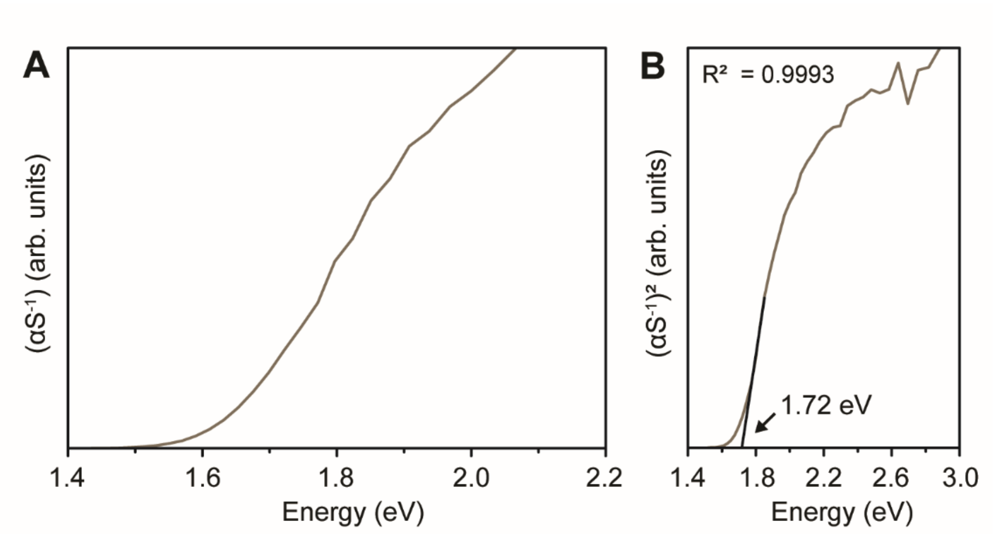

Figure S17. (**A**) Diffuse reflectance spectrum for (Hepz)[Pb_2Br_6]. The diffuse reflectance data were transformed using the Kubelka–Munk function (α and S are the pseudo-absorption and scattering coefficients, respectively). (**B**) Tauc plot for (Hepz)[Pb_2Br_6].

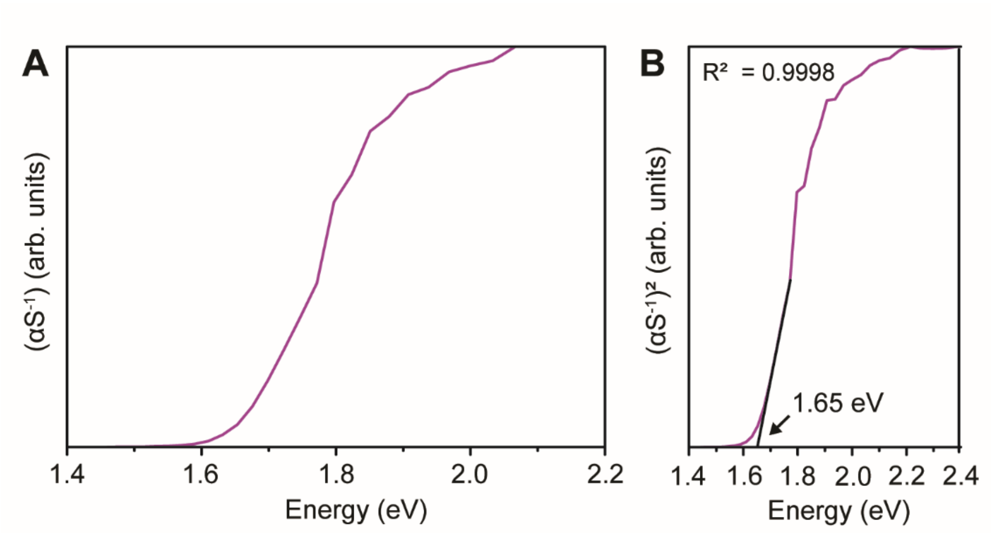

Figure S18. (**A**) Diffuse reflectance spectrum for (Hepm)[Pb_2I_6]. The diffuse reflectance data were transformed using the Kubelka–Munk function (α and S are the pseudo-absorption and scattering coefficients, respectively). (**B**) Tauc plot for (Hepm)[Pb_2I_6].

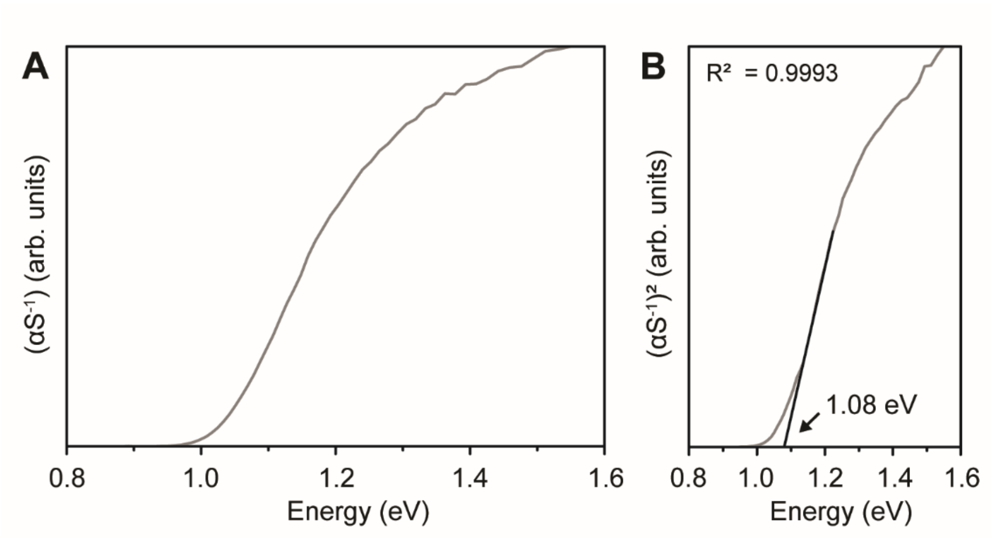

Figure S19. (**A**) Diffuse reflectance spectrum for (Hepm)[Sn_2I_6]. The diffuse reflectance data were transformed using the Kubelka–Munk function (α and S are the pseudo-absorption and scattering coefficients, respectively). (**B**) Tauc plot for (Hepm)[Sn_2I_6].

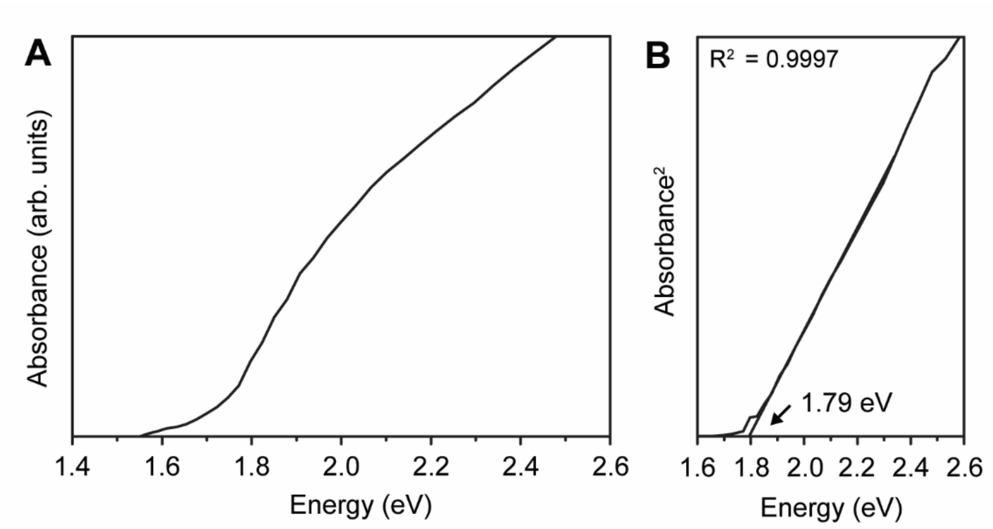

Figure S20. (**A**) Absorbance spectrum for $(Hepz)_{0.01}(Hepm)_{0.99}[Pb_2Br_6]$. (**B**) Tauc plot for $(Hepz)_{0.01}(Hepm)_{0.99}[Pb_2Br_6]$.

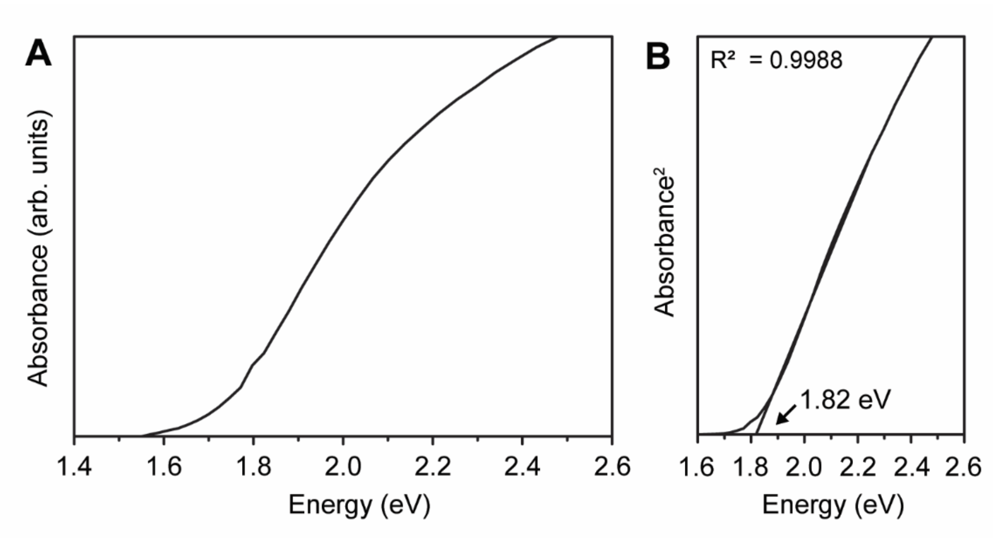

Figure S21. (**A**) Absorbance spectrum for $(Hepz)_{0.02}(Hepm)_{0.98}[Pb_2Br_6]$. (**B**) Tauc plot for $(Hepz)_{0.02}(Hepm)_{0.98}[Pb_2Br_6]$.

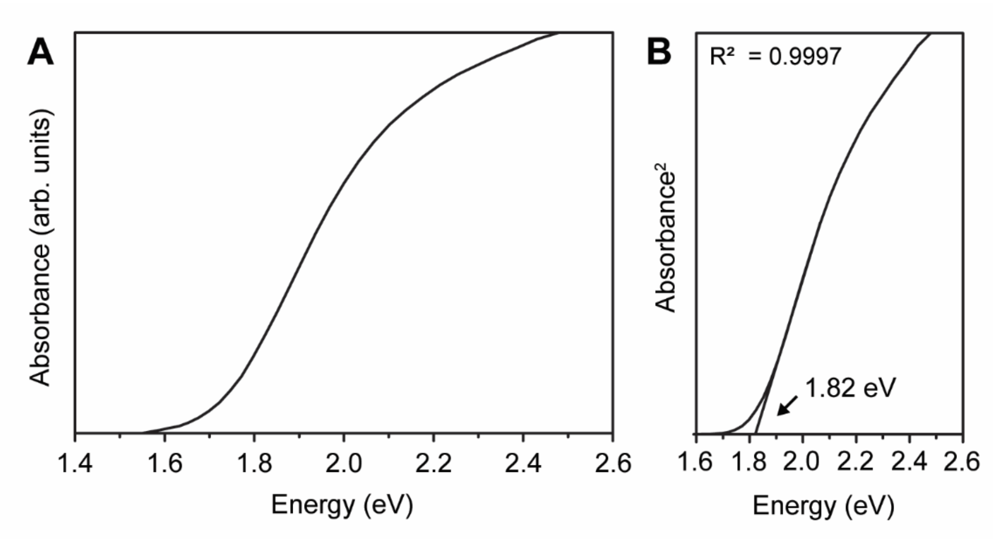

Figure S22. (**A**) Absorbance spectrum for $(Hepz)_{0.05}(Hepm)_{0.95}[Pb_2Br_6]$. (**B**) Tauc plot for $(Hepz)_{0.05}(Hepm)_{0.95}[Pb_2Br_6]$.

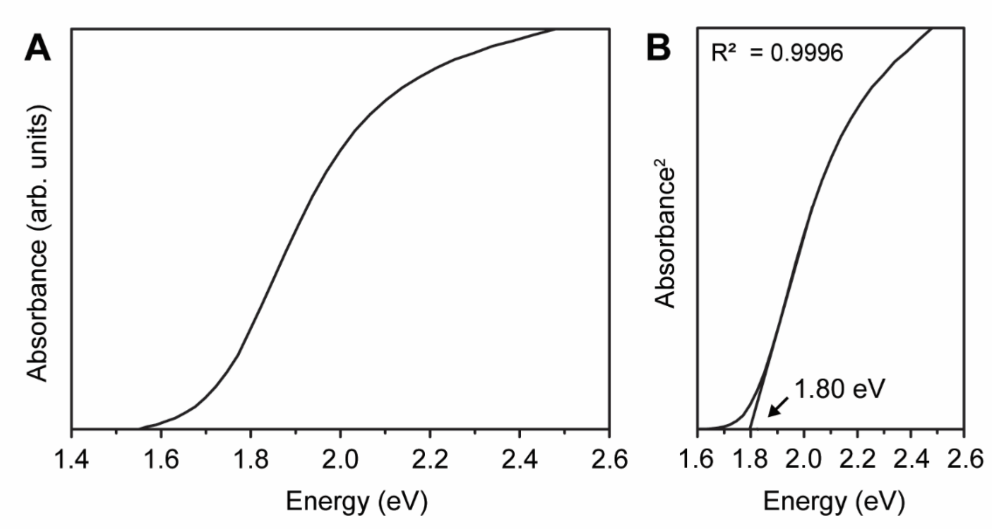

Figure S23. (**A**) Absorbance spectrum for $(Hepz)_{0.10}(Hepm)_{0.90}[Pb_2Br_6]$. (**B**) Tauc plot for $(Hepz)_{0.10}(Hepm)_{0.90}[Pb_2Br_6]$.

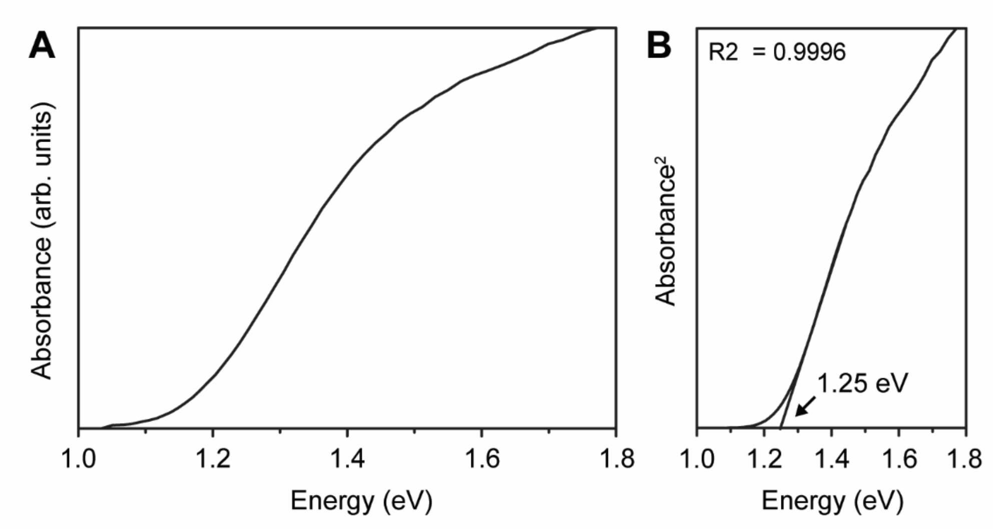

Figure S24. (**A**) Absorbance spectrum for $(Hepz)_{0.01}(Hepm)_{0.99}[Pb_2Br_{0.06}I_{5.94}]$. (**B**) Tauc plot for $(Hepz)_{0.01}(Hepm)_{0.99}[Pb_2Br_{0.06}I_{5.94}]$.

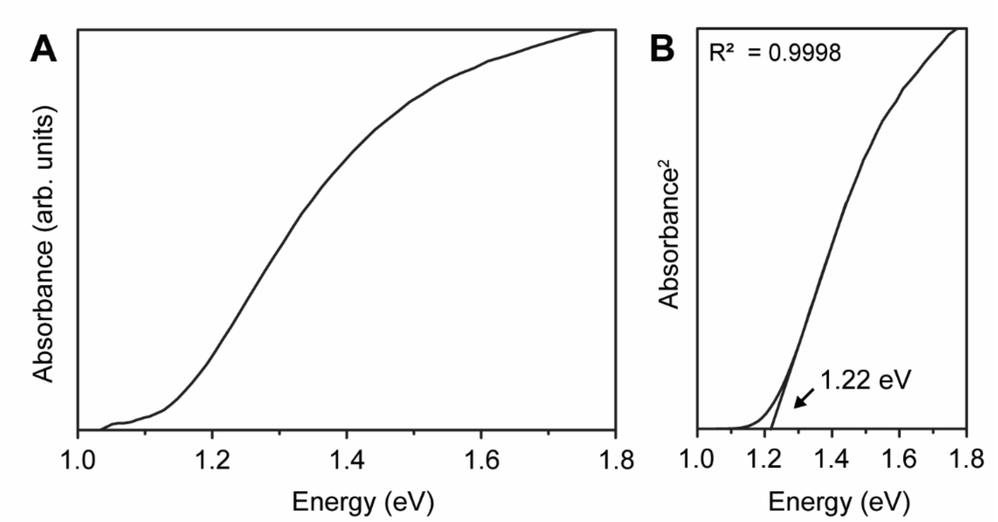

Figure S25. (**A**) Absorbance spectrum for $(Hepz)_{0.02}(Hepm)_{0.98}[Pb_2Br_{0.12}I_{5.88}]$. (**B**) Tauc plot for $(Hepz)_{0.02}(Hepm)_{0.98}[Pb_2Br_{0.12}I_{5.88}]$.

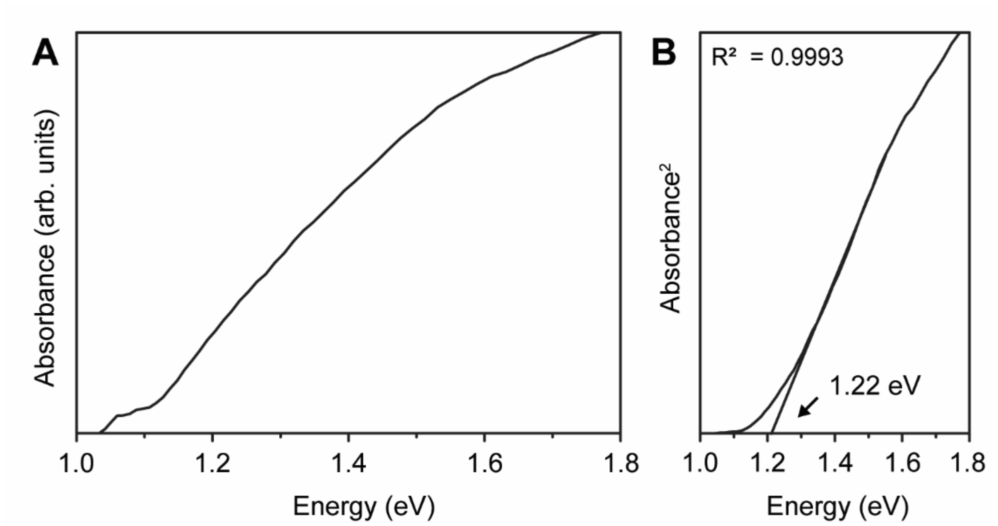

Figure S26. (**A**) Absorbance spectrum for $(Hepz)_{0.05}(Hepm)_{0.95}[Pb_2Br_{0.30}I_{5.70}]$. (**B**) Tauc plot for $(Hepz)_{0.05}(Hepm)_{0.95}[Pb_2Br_{0.30}I_{5.70}]$.

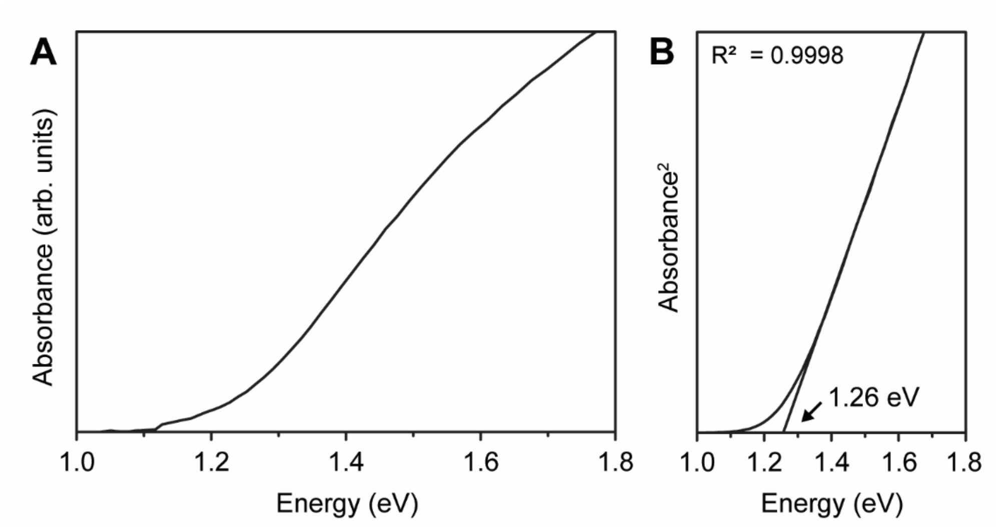

Figure S27. (**A**) Absorbance spectrum for $(Hepz)_{0.10}(Hepm)_{0.90}[Pb_2Br_{0.60}I_{5.40}]$. (**B**) Tauc plot for $(Hepz)_{0.10}(Hepm)_{0.90}[Pb_2Br_{0.60}I_{5.40}]$.

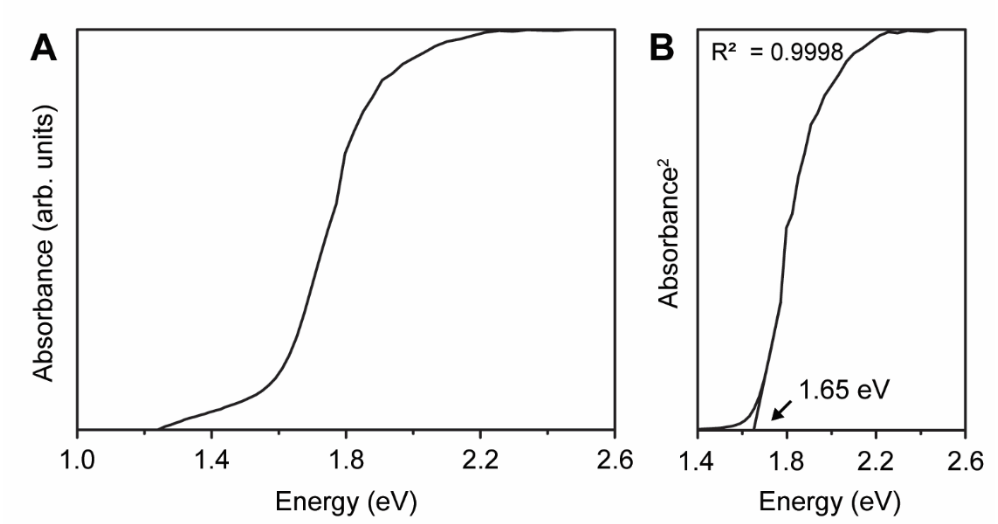

Figure S28. (**A**) Absorbance spectrum for $(Hepm)[Pb_2Br_{0.06}I_{5.94}]$. (**B**) Tauc plot for $(Hepm)[Pb_2Br_{0.06}I_{5.94}]$.

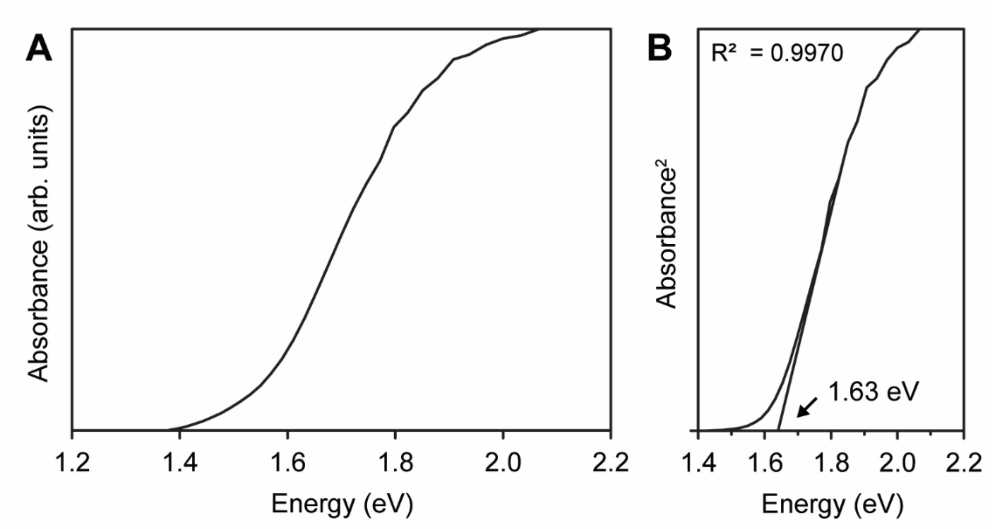

Figure S29. (**A**) Absorbance spectrum for (Hepm)[$Pb_2Br_{0.30}I_{5.70}$]. (**B**) Tauc plot for (Hepm)[$Pb_2Br_{0.30}I_{5.70}$].

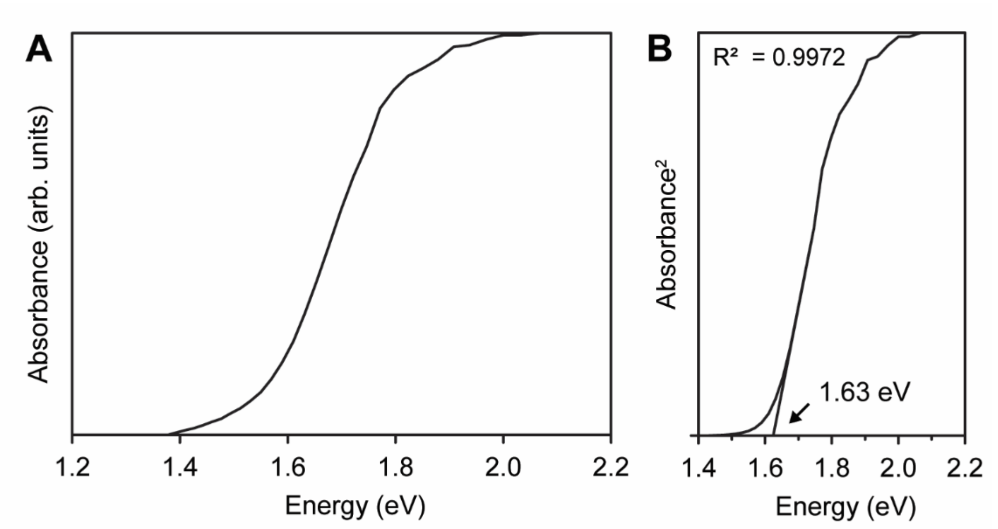

Figure S30. (**A**) Absorbance spectrum for (Hepm)[$Pb_2Br_{0.60}I_{5.40}$]. (**B**) Tauc plot for (Hepm)[$Pb_2Br_{0.60}I_{5.40}$].

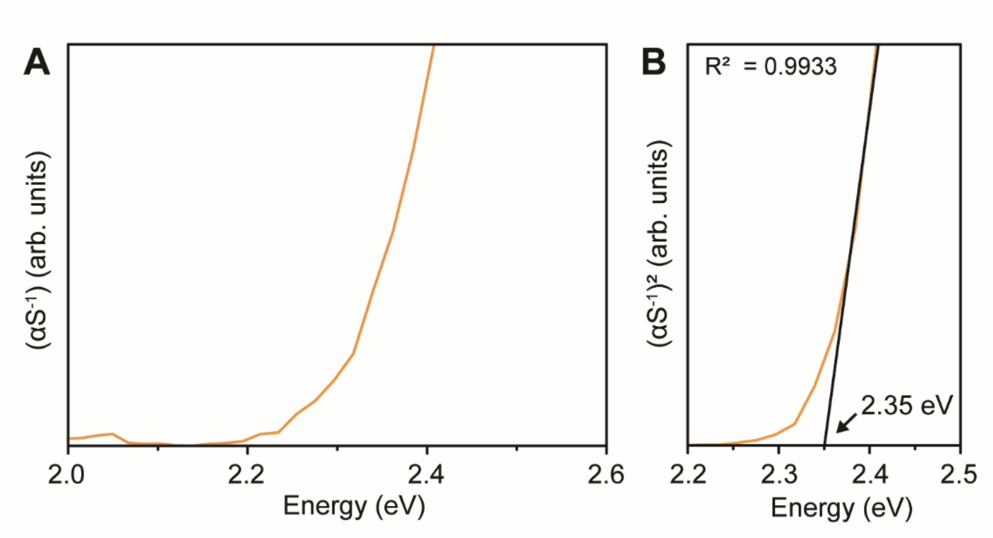

Figure S31. (**A**) Absorbance spectrum for inkjet printed film of (Hepm)[Pb_2Br_6]. (**B**) Tauc plot for the inkjet printed film of (Hepm)[Pb_2Br_6].

Table S4. Calculated optical gaps (E_g, eV) for (Hepm)[B_2X_6] (B–X = Pb–Br, Pb–I, Sn–I) and (Hepz)[Pb_2Br_6] using two different Tauc plot calculations.

	A^2 and $(\alpha S^{-1})^2$ Tauc Plot	$(A\cdot h\upsilon)^2$ and $(\alpha S^{-1}\cdot h\upsilon)^2$ Tauc Plot
(Hepm)[Pb_2Br_6]	2.30(5)	2.30(5)
(Hepz)[Pb_2Br_6]	1.70(5)	1.70(5)
(Hepm)[Pb_2I_6]	1.60(5)	1.65(5)
(Hepm)[Sn_2I_6]	1.00(5)	1.05(5)

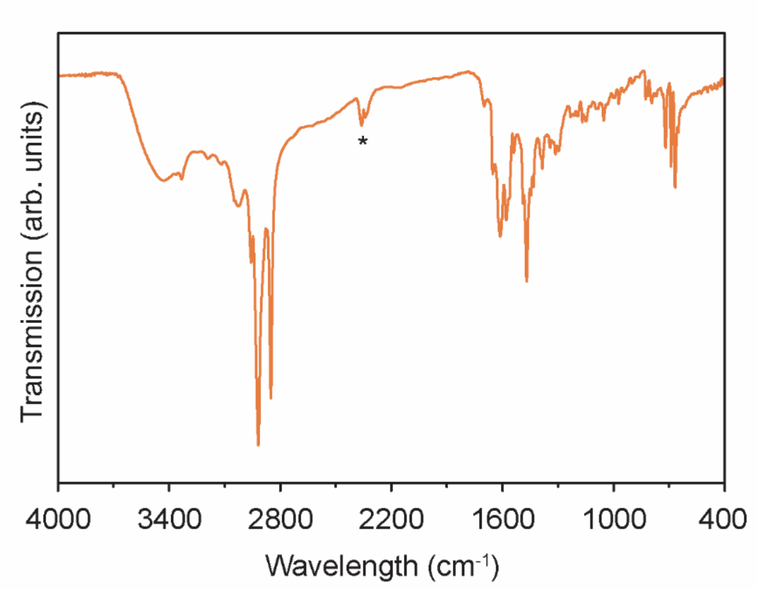

Figure S32. Infrared spectrum for (Hepm)[Pb_2Br_6] at ambient pressure. The * indicates the peaks corresponding to O=C=O stretches from ambient CO_2.

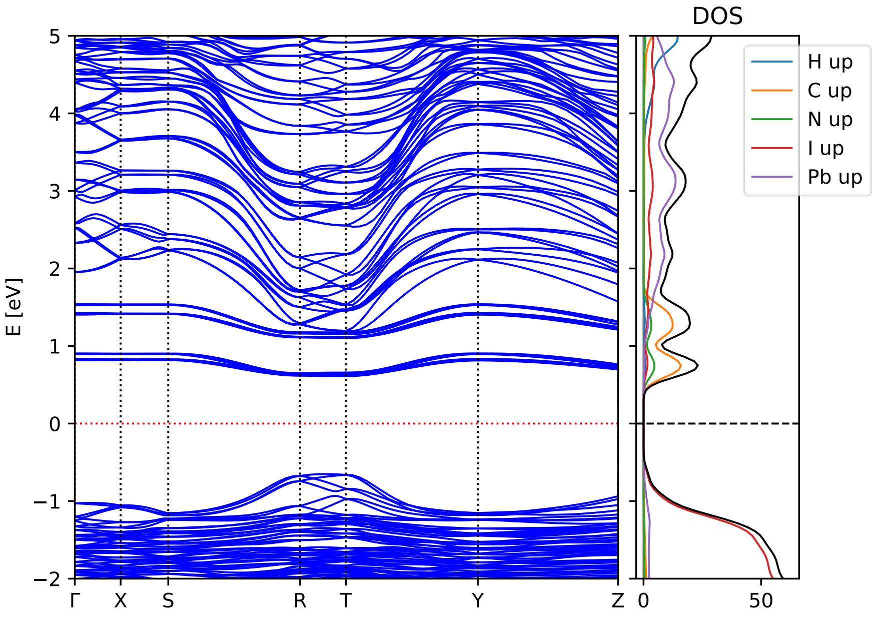

Figure S33. Calculated electronic band structures and density-of-states (DOS) for (Hepm)[Pb_2I_6] using the fhi-aims code with the HSE06 hybrid functional, including spin-orbit effects.

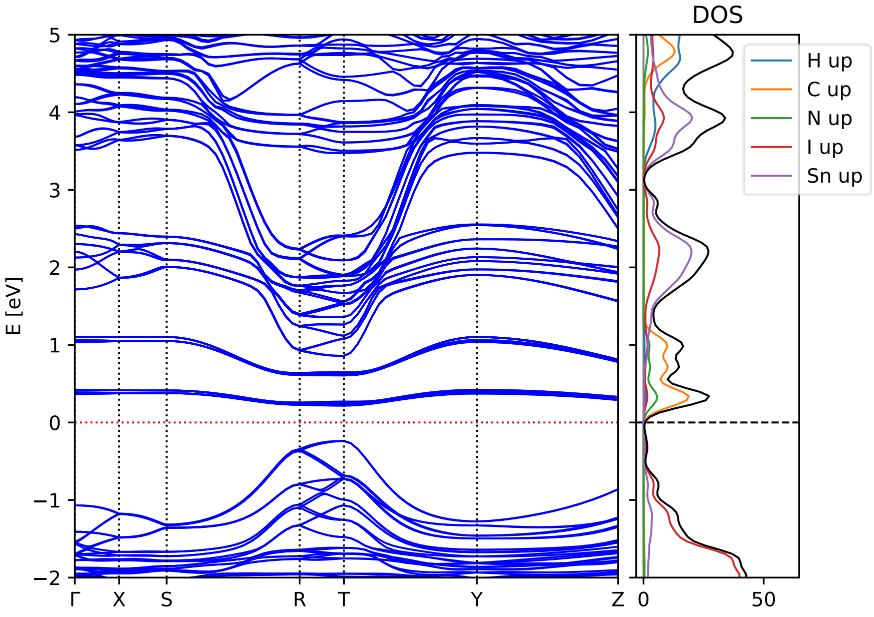

Figure S34. Calculated electronic band structures and density-of-states (DOS) for (Hepm)[Sn_2I_6] using the fhi-aims code with the HSE06 hybrid functional, including spin-orbit effects.

**Table S5.** Calculated energy in eV of the molecular orbitals of $Hepz^{2+}$ and $Hepm^{2+}$ using HSE06 functional.		
	**$Hepz^{2+}$**	**$Hepm^{2+}$**
HOMO	-18.06	-18.10
LUMO	-13.63	-13.10
LUMO +2	-11.93	-12.52

**Table S6.** HSE06-calculated hole effective masses for (Hepm)[Pb_2Br_6] and (Hepm)[Pb_2Br_6] in electron mass units (m_e).		
Direction	[100]	[001]
(Hepz)[Pb_2Br_6]	1.00	0.26
(Hepm)[Pb_2Br_6]	1.27/1.32	0.28

**Table S7.** Comparison of the experimental optical and HSE06-calculated optical gaps (E_g)		
	**Calculated E_g (eV)**	**Experimental E_g (eV)**
(Hepm)[Pb_2Br_6]	1.70	2.30(5)
(Hepz)[Pb_2Br_6]	1.00	1.70(5)
(Hepm)[Pb_2I_6]	1.20	1.60(5)
(Hepm)[Sn_2I_6]	0.40	1.00(5)

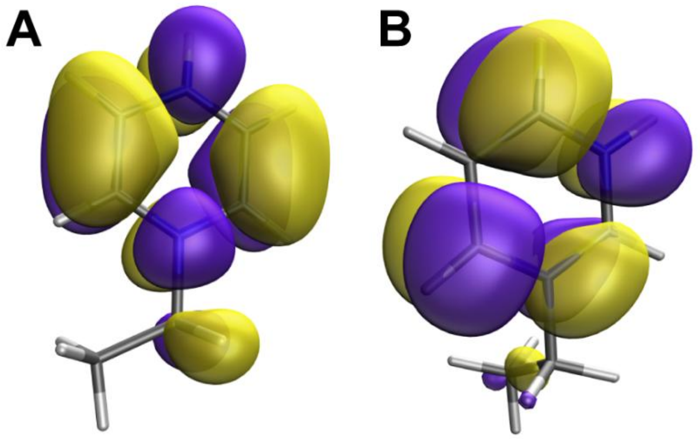

Figure S35. LUMO molecular orbitals for (**A**) $Hepz^{2+}$ and (**B**) $Hepm^{2+}$ calculated with the HSE06 functional using an isosurface value of 0.05 e Å^{-3}.

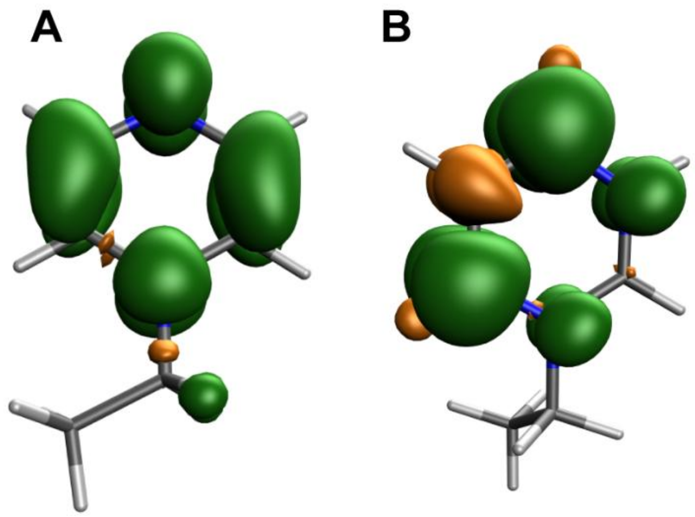

Figure S36. Spin density plots for (**A**) Hepz$^{\bullet+}$ and (**B**) Hepm$^{\bullet+}$ calculated with the HSE06 functional using an isosurface value of 0.02 e Å^{-3}. Green and orange lobes indicate positive and negative values, respectively. In **A**, the LUMO is delocalized in the whole ring (see Figure S36). However, the negative spin density in **B** appears in the C atom coordinated with the C atoms with the largest spin density due to the spin polarization.

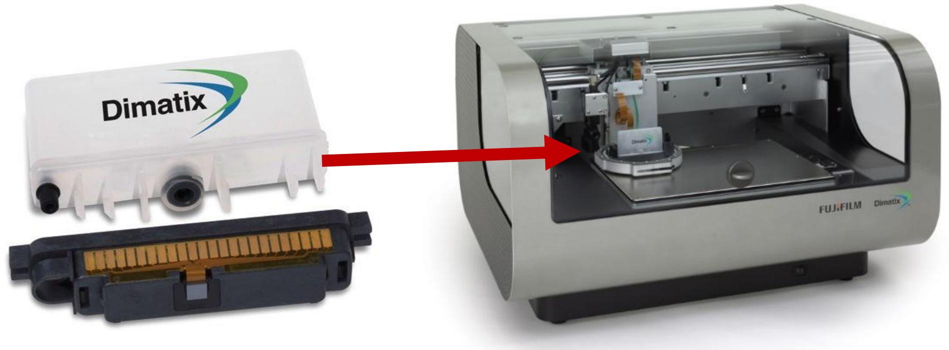

Figure S37. Fujifilm Dimatix cartridge with 21 µm diameter nozzles

Table S8. Summary of the ink rheology parameters

**Viscosity (η)**	g cm^{-1} s^{-1}	0.021
**Surface tension (ϒ)**	dyn cm^{-1}	26
**Density (ρ)**	g cm^{-3}	1.1
**Nozzle diameter (α)**	cm	0.0021
**Velocity (ν)**	cm s^{-1}	175

**Table S9.** Summary of the ink rheology numbers under different fluid velocities			
**Velocity (cm s^{-1})**	**Re**	**We**	***Z***
50	3.10	0.078	11.09
200	7.22	0.43	11.01
500	10.31	0.87	11.05
700	13.41	1.47	11.06

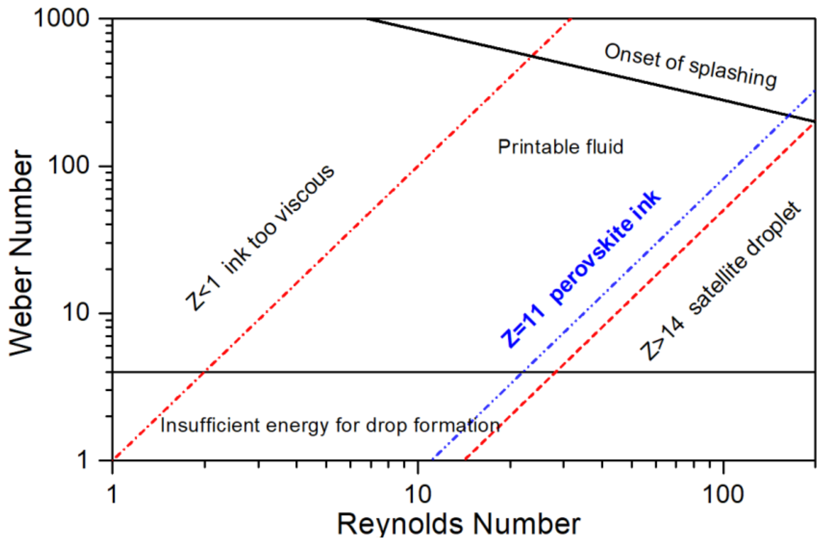

Figure S38. Weber number vs. Reynold number plot used to optimize the composition of the perovskite ink.

**Table S10.** Summary of the ink printing parameters		
**Voltage amplitude**	V	25
**Drop speed**	m s^{-1}	4
**Nozzle temperature**	^{o} C	--
**Drop spacing**	µm	30
**Printhead – Substrate**	µm	400
**Substrate temperature**	^{o} C	20
